# Targeting CAMKII to reprogram tumor-associated macrophages and inhibit tumor cells for cancer immunotherapy with an injectable hybrid peptide hydrogel

**DOI:** 10.7150/thno.42385

**Published:** 2020-02-10

**Authors:** Xiaomeng Dai, Jingshu Meng, Suke Deng, Lingling Zhang, Chao Wan, Lisen Lu, Jing Huang, Yan Hu, Zhanjie Zhang, Yan Li, Jonathan F. Lovell, Gang Wu, Kunyu Yang, Honglin Jin

**Affiliations:** 1 Cancer Center, Union Hospital, Tongji Medical College, Huazhong University of Science and Technology, Wuhan 430022, China; 2 Department of Chemical and Biological Engineering, University at Buffalo, State University of New York. Buffalo, New York 14260, USA

**Keywords:** Hydrogel, cancer immunotherapy, tumor-associated macrophages, anti-PD-1, CAMKII

## Abstract

Simultaneously targeted treatment of tumor cells and their surrounding growth-supporting immune cells is a promising strategy to reshape immunosuppressive tumor microenvironment (TME) and potentiate host innate and adaptive antitumor immune responses.

**Methods**: We designed a series of melittin-(RADA)_n_ hybrid peptide sequences with varying self-assembling motifs of RADA and screened out a melittin-(RADA)_6_ peptide that has an optimal gel-formation ability and *in vitro* antitumor activity.

**Results**: The formed melittin-(RADA)_6_ (MR_52_) hydrogel scaffold could be loaded with a specific Ca^2+^/calmodulin-dependent protein kinase II (CAMKII) inhibitor, KN93, originally found to have both direct tumoricidal activity and macrophages-reprogramming ability, for potent immunotherapy against melanoma and hepatoma ascites in mice models. Our MR_52_ hydrogel has an interweaving nanofiber-like structure, possesses direct antitumor and controlled drug release properties*,* and promotes the enhanced intracellular uptake of loaded cargo. Compared to free KN93, the MR_52_-KN93 hydrogel (MRK) improved the killing effects and levels of immunogenic cell death (ICD) on tumor cells significantly. Due to the dual role of KN93, the injection of the MRK hydrogel retarded the growth of subcutaneous melanoma tumors dramatically and resulted in a high number of mature dendritic cells of draining lymph nodes, significantly enhancing the portion of cytotoxic T cells and reduced number of M2-like tumor-associated macrophages (TAMs) in tumors. Using a mouse model of malignant ascites (MAs), where traditional therapy was ineffective, we demonstrated that the MRK hydrogel treatment offered a significantly prolonged survival compared to controls. Following treatment with the MRK hydrogel, macrophages had elevated programmed cell death protein ligand-1 (PD-L1) expression, promising follow-up combined anti-PD-1 therapy that confers a cure rate of approximately 30% against MAs in mice models.

**Conclusion**: Thus, the MRK hydrogel may serve as a prospective platform for antitumor applications.

## Introduction

Understanding the complicated tumor cell-immune cell communication networks is pivotal to constructing novel therapeutic strategies against cancers 1-3. A large number of studies have confirmed that malignant tumor cells can recruit a variety of inhibitory immune cells into the tumor microenvironment (TME) by secreting cytokines and chemokines (such as CCL2 and CCL3), including tumor-associated macrophages (TAMs), regulatory T cells (Regulatory T cells, Tregs), and bone marrow-derived inhibitory cells (Myeloid-derived suppressor cells, MDSCs)^4^-^7^. Among these immuno-suppressive cells, TAMs are major components of the TME and there is, at present, strong clinical evidence that links TAMs with cancer progression and metastasis 8-10. There are two main polarization types of TAMs, classical activated macrophages (M1) and alternatively activated macrophages (M2). It has been confirmed that IFN-γ, LPS and other pro-inflammatory factors in TME induce TAMs to M1 polarization, which can synthesize and secrete cytotoxic molecules such as reactive oxygen species (ROS), TNFα and NO, using HLA-DR, CD80, CD86 and other molecules as the primary surface markers. Notably, M1-TAMs have the ability to scavenge foreign antigens and kill tumor cells. On the other hand, M2 polarization is usually induced by IL-4, IL-13, and IL-10 in TME, using CD163, CD206, CD369 and other molecules as the primary surface markers. M2 macrophages are further subdivided in M2a (after exposure to IL-4 or IL-13), M2b (immune complexes in combination with IL-1β or LPS) and M2c (IL-10, TGF-β or glucocorticoids) 11. As the main TAMs in TME, M2-TAMs can synthesize anti-inflammatory cytokines such as IL-10 and TGF, inhibit antitumor immune responses and promote tumor growth 6, 9, 12. M2-TAMs can also be transformed into M1-TAMs to play a tumoricidal role when stimulated by some external stimuli 13, which provides an important opportunity for improved therapeutic approaches against tumors. How to awaken microenvironmental M2-TAMs to make their differentiation into M1-TAMs has increasingly become a new hot spot in the field of cancer immunotherapy 1, 14, 15. Therefore, efficient cancer immunotherapy requires not only the therapeutic effects on tumor cells but also the promotion of TAMs reprogramming and differentiation into M1-TAMs.

Related studies have shown that Ca^2+^/calmodulin-dependent protein kinase II (CAMKII), a multi-functional serine/threonine kinase, plays oncogenic roles in leukemia, lymphocyte and hepatocellular carcinoma through the mechanisms of stabilizing the level of classical oncoprotein c-Myc by inhibiting its ubiquitination degradation 16-18. CAMKII can also promote the necrosis of atherosclerotic plaques by inhibiting the burial of macrophages 19. However, there is no corresponding study on whether CAMKII plays a related role in the reprogramming of macrophages. As a specific inhibitor of CAMKII, KN93 can inhibit the activity of CAMKII effectively. Due to the close link between CAMKII and macrophages, we therefore, hypothesize that KN93 has both direct tumoricidal activity and macrophages-reprogramming capability. However, the *in vivo* application of these small molecule inhibitors is often encountered with inefficient intracellular uptake and short circulating time, as well as low levels of induction of immunogenic cell death (ICD) to activate subsequent host adaptive immunity. Thus, new delivery approaches are required for KN93 to maximize its direct anti-tumor and immune-regulating effects.

One solution to fulfill this goal is the utilization of peptide hydrogels, such as RADA16-I (RADARADARADARADA), EAKl6 (AEAEAKAKAEAEAKAK) and KLD-12 (KLDLKLDLKLDL), obtained by designer amphiphilic self-assembling short peptides that have been widely used in biomedical application, including tissue engineering, regenerative medicine and tumor therapy 20. Due to their immense drug loading ability, high payload encapsulation efficiency, strong sustained-release capacity and excellent biocompatibility, a variety of peptide hydrogels have been designed for chemotherapy, chemo-immunotherapy and immunotherapy 21-27. These antitumor peptide hydrogels are composed basically of a hydrogel scaffold, therapeutic drugs or immune-regulating molecules incorporated. However, in most of these platforms, the hydrogel scaffold itself only provides sustained release of loaded antitumor agents, but has no additional functions to facilitate cancer therapy. In this study, we provided an original report that KN93 exhibits both direct antitumor and macrophages-reprogramming promoting ability. To further potentiate this functional effects, we designed a hybrid peptide hydrogel sequence composed of (RADA)_6_, linker peptides, and melittin, a 26-amino-acid polypeptide contributing the major peptide component of bee venom, which can not only reduce the hemolytic toxicity of melittin, but also give it the ability to kill tumor cells as our previous study indicated^28^, ^29^. The formed melittin-(RADA)_6_ (MR_52_) hydrogel scaffold, with optimal abilities in gel-formation and *in vitro* antitumor activity, was loaded efficiently with KN93 (100%), improving its intracellular and pharmacokinetic behavior significantly. The resulting MR_52_-KN93 hydrogel (MRK) elicited a potent immunotherapy against mice models of melanoma, and more importantly in malignant ascites (MAs), an extensive peritoneal metastasis where traditional therapy was inferior. Due to the observation that MRK hydrogel treatment induces the elevated programmed cell death protein ligand-1 (PD-L1) expression in macrophages, we demonstrated further the combination of MRK hydrogel with anti-PD-1 therapy provides ablation effects against MA in mouse models (**Graphical Abstract**).

## Results

### Synthesis, characterization, and *in vitro* antitumor effects of the MRK hydrogel

**Figure 1:**
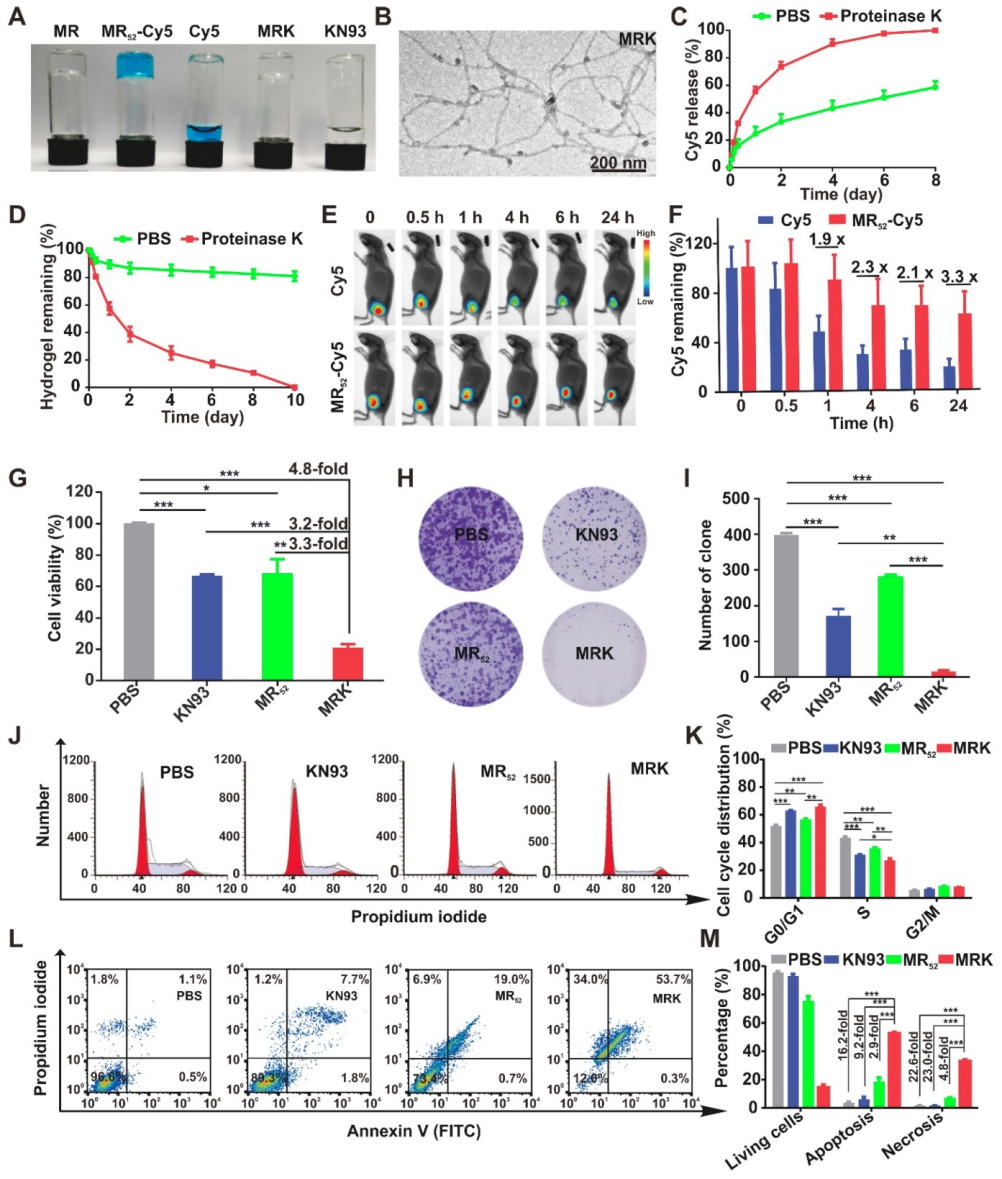
** Synthesis, characterization, and *in vitro* antitumor effects of the MRK hydrogel. (A)** Photographs of MR_52_ hydrogels or MR_52_ hydrogels loaded with various agents. **(B)** The representative TEM image of the MRK hydrogel. Scale bar, 200 nm. **(C)** Comparison of the release rate of Cy5 from the MR_52_-Cy5 hydrogel in the presence or absence of proteinase K. **(D)** Comparison of the MR_52_ hydrogel release rate in the presence or absence of proteinase K. **(E)** The NIR fluorescence imaging analysis of the distribution of the MR_52_-Cy5 hydrogel and free Cy5 *in vivo*. The subcutaneous implantation was performed at the indicated time points. **(F)** Quantitative data of the NIR fluorescence imaging results, Data are presented as the mean ± SEM (n = 3). **(G)** Cell viability measurement using the CCK-8 assay. **(H, I)** Clone formation assay (left panel: representative images of cell clones treated with PBS, KN93, and MR_52_, or MRK hydrogel; right panel: quantification of cell clones; n = 3). **(J)** Representative images of the cell cycle induced by PBS, KN93, MR_52_, or MRK hydrogel per the flow cytometry analyses. **(K)** Quantitative data from **(J)**. Data are presented as the mean ± SEM (n = 4). **(L-M)** Flow cytometry analyses of cell apoptosis induced by PBS, KN93, MR_52_, or MRK hydrogel. Data are presented as the mean ± SEM (n = 3).

To prepare a proper melittin-encapsulated hydrogel scaffold, we designed three kinds of melittin-containing fusion peptide based on the length of RADA, melittin-(RADA)_4_, melittin-(RADA)_6_ and melittin-(RADA)_8_, denoted as MR_44_, MR_52_, MR_60_, respectively, according to the total peptide length. When loaded with the fluorescence dye Cy5, the MR_52_ and MR_60_ peptides gelated quickly, while MR_44_ did not form a hydrogel in the presence of 0.9% NaCl (w/w) (**Figure S1A**). To evaluate the influence of RADA length on *in vitro* antitumor effects, we treated melanoma cells B16F10 with MR_52_ and MR_60_ under various concentrations. We found that the MR_52_ hydrogel had a superior killing effect on B16F10 than MR_60_ (**Figure S1B**), with the half-maximal inhibitory concentrations (IC50) of 15.5 *μ*M and 49.2 *μ*M, respectively. Also, MR_52_ is much easier for peptide synthesis than MR_60_ due to the absence of eight amino acids. Therefore, these results suggest that MR_52_ is a desired melittin-encapsulated hydrogel scaffold with optimal gel-formation ability and antitumor activity. As shown in **Figure 1A**, the MR_52_ hydrogel platform was well-suited for the loading of Cy5 and CAMKII inhibitor KN93. Strikingly, the loading efficiency of these cargoes into MR_52_ reached almost 100%, which may prove beneficial for drug delivery. We analyzed the size and shape of MRK hydrogel using transmission electron microscopy (TEM). The results indicated that MR and MRK hydrogel self-aggregated into networks of interweaving nanofiber structure with diameters of 11.0 ± 0.5 and 13.0 ± 0.4 nm, respectively (**Figure 1B, Figure S2**). When the strain constant was maintained at 0.1%, both the storage modulus (G′) and loss modulus (G″) of the MRK hydrogel were slightly dependent on angular frequency (0.1-100 rad/s), indicating the stiffness and stability of MRK hydrogel (**Figure S3**). To study the release rate of KN93 from the MRK hydrogel in solutions and mice models, we used MR-Cy5 to mimic MRK due to the similar molecular weight and water solubility between KN93 and Cy5; the ease of visualization and quantification was based on the near-infrared (NIR) optical properties of Cy5. As shown in **Figure 1C,** MR_52_-Cy5 had a release rate of 24.6% at 24 h, 33.4% at 48 h, and 43.2% at 96 h in the absence of proteinase K, which can digest peptides and proteins. In contrast, in the presence of proteinase K, release rates were 55.5% at 24 h, 73.4% at 48 h, and 90.0% at 96 h, suggesting that the presence of proteinase K accelerated the release rate of Cy5 from MR_52_-Cy5 significantly. Furthermore, we performed High Performance Liquid Chromatography (HPLC) to measure the release of KN93 from the MR_52_ hydrogel *in vitro* in the presence or absence of proteinase K. The release rates were very similar to Cy5 release from the MR_52_ hydrogel (**Figure S4**). At the same time, we examined the release of the MR_52_ hydrogel backbone peptide. According to **Figure 1D**, on day 10, 81% of the MR_52_ hydrogel remained unassimilated in the absence of proteinase K. In sharp contrast, the MR_52_ hydrogel had been entirely digested in the same period in the presence of proteinase K, suggesting an excellent biodegradability of the MR_52_ hydrogel scaffold. Additionally, we compared the release profile of the MR-Cy5 hydrogel and free Cy5 in mice after subcutaneous injection and detection by the real-time NIR fluorescence imaging. According to **Figure 1E-F**, the fluorescence signals started to differentiate 1 h after injection. The signals of the MR_52_-Cy5 hydrogel group were 1.9, 2.3, 2.1 and 3.3-fold more detectable than those in the free Cy5 group at 1 h, 4 h, 6 h and 24 h, respectively, and these signals remained highly detectable in the MR_52_-Cy5 group on day 9, whereas, they were not detectable in the free Cy5 group (**Figure S5**). These results indicate that the MR_52_ hydrogel can evidently promote the sustained release of the loaded cargoes. Based on our findings, we successfully developed a biodegradable, sustained-releasable, and KN93 and melittin-loaded peptide hydrogel.

The abnormal high expression of CAMKII and its tumor-promoting functions have been verified in tumors, such as melanoma and hepatoma 17, 18. To investigate the potential role of the MRK hydrogel in cancer immunotherapy, mice melanoma B16F10 and hepatoma H22 cells were used in this study. The cytotoxicity of KN93 and the MR_52_ and MRK hydrogels against B16F10 cells were examined using the CCK-8 assay and clone formation. Results showed that the IC50 for KN93 was about 12 *μ*M (**Figure S6**). As shown in **Figure 1G**, the MRK hydrogel group exhibited noticeable cell inhibition, with 4.8-fold, 3.2-fold, and 3.3-fold more reductions in cell viability, compared to the PBS, KN93, MR_52_ groups, respectively. To evaluate whether this combination has synergistic effects, we calculated the combination index (CI) through the Chou-Talalay method, and the CI was 0.44 (CI = 1 indicates additive effect, CI < 1 indicates synergism, CI > 1 indicates antagonism). In line with the results of the CCK-8 assay, clone formation assay also demonstrated significantly fewer clones than the other groups (**Figure 1H-I**). These results indicate an apparent enhanced inhibitory effect of the MRK hydrogel against B16F10 cells. Next, we unearthed the underlying mechanism for MRK-enhanced cell death by flow cytometry to analyze changes in cell cycle and apoptosis.

The cell cycle analysis showed that the MRK group had a significantly lower cell portion during the S phase and an increased amount of cells distributed during the G0/G1 phase, suggesting that the MRK hydrogel may have promoted an inhibitory effect on DNA synthesis at the S phase, which in turn caused a considerable growth suppression against B16F10 (**Figure 1J-K**). Moreover, as shown in **Figure 1L-M**, the percentages of cell apoptosis in the MRK hydrogel group were 16.2, 9.2, and 2.9 times those in the groups of PBS, KN93, and MR_52_, respectively. We found that the MRK hydrogel caused 22.6-fold, 23.0-fold, and 4.8-fold increases in cell necrosis rates, compared to the PBS, KN93, and MR_52_ group, respectively. Collectively, these results revealed that MRK exerts *in vitro* antitumor effects mainly through the inhibition of DNA synthesis during the S phase and *via* the induction of cell apoptosis and necrosis.

### The MRK hydrogel induces ICD and facilitates intracellular uptake of loaded cargo in vitro

**Figure 2:**
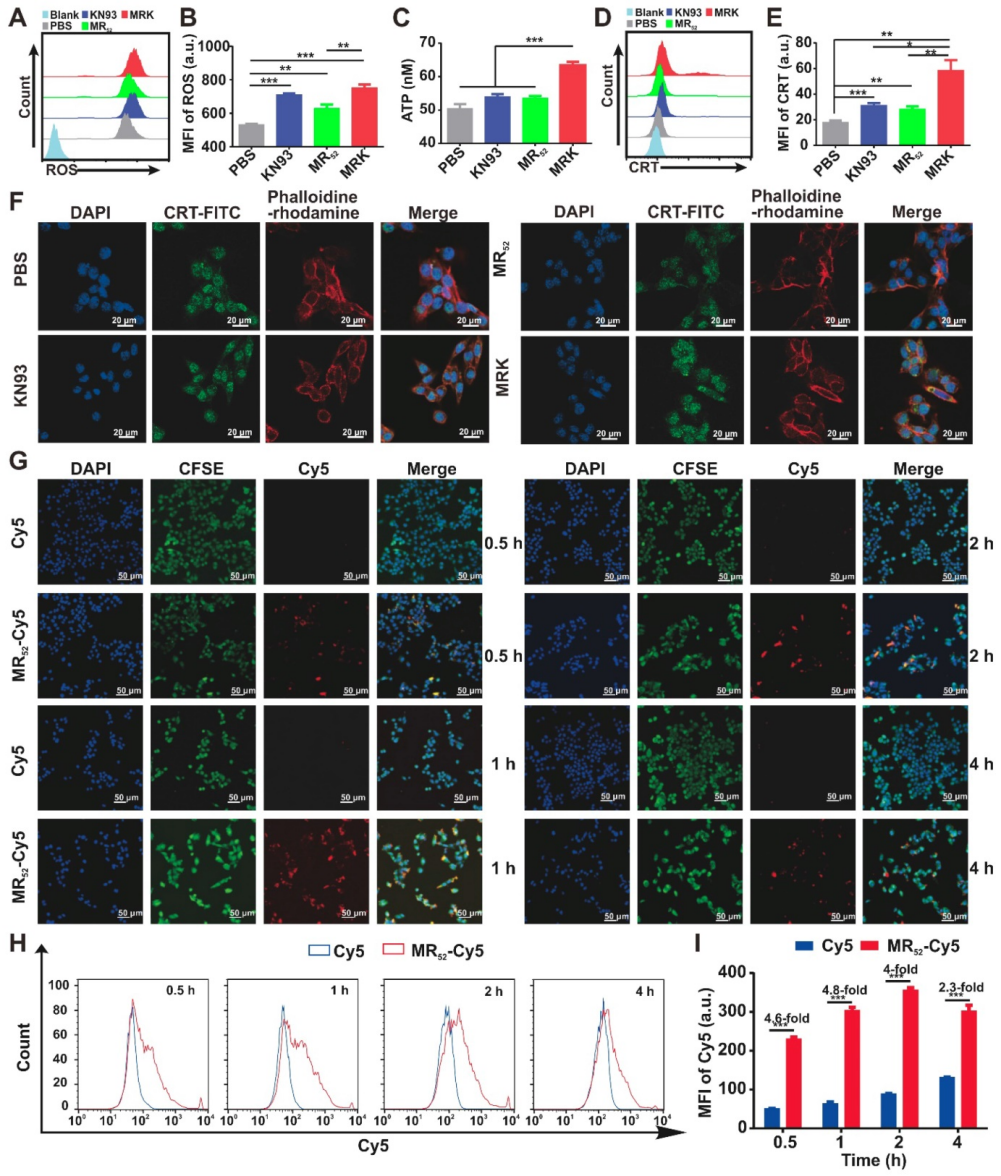
**The MRK hydrogel induces ICD and facilitates intracellular uptake of loaded cargo *in vitro***. **(A, B)** Flow cytometry measurement of ROS induced by PBS, KN93, MR52, or MRK hydrogel. Data are presented as the mean ± SEM (n = 4). **(C)** ATP levels in the B16F10 cells treated with indicated compounds were measured using the multiscan spectrum. Data are presented as mean ± SEM (n = 4). **(D, E)** Flow cytometry analyses of CRT expression on the cell membrane surface. Data are presented as the mean ± SEM (n = 4). **(F)** Representative immunofluorescence staining images of CRT expression (green) on the surface of B16F10 cells after various treatments. Phalloidine-rhodamine (red) indicates the cytoskeleton (scale bar, 20 µm). **(G)** Representative immunofluorescence images of the intake of Cy5 in B16F10 cells treated with MR_52_-Cy5 or free Cy5 at the indicated time points (scale bar, 50 *µ*m). **(H, I)** Flow cytometry analyses of Cy5 uptake in B16F10 cells after MR_52_-Cy5 or free Cy5 treatments at indicated time points. Data are presented as the mean ± SEM (n = 3).

We next investigated whether MRK hydrogel could enhance ICD levels in B16F10 cells. Cumulative evidences have suggested that an increase in ROS levels in the cytoplasm can cause ICD 30. As expected, the MRK hydrogel group had augmented cytosolic ROS levels (**Figure 2A-B**), suggesting that the death of B16F10 caused by the MRK hydrogel may be associated with ICD. Thus, we examined the danger-associated molecular patterns (DAMPs) of B16F10 cells after treatment with KN93, MR_52_ or MRK hydrogel. The results showed that the expression of extracellular calreticulin (CRT) and the levels of adenosine triphosphate (ATP) released in the medium increased significantly in the MRK hydrogel group than those of the other groups (**Figure 2C-E**). Consistent with the results of flow cytometry analysis, confocal microscopy also confirmed that the MRK hydrogel group had more focus amount of CRT expression on the membrane of B16F10 cells than the other groups (**Figure 2F**). Altogether, the MRK hydrogel can promote the induction of ICD levels in B16F10 cells. Given that KN93 can also block K+ channels independently of CAMKII inhibition, and cardiac glycosides have been published by Guido Kroemer *et al*. as potent inducers of ICD *via* inhibiting sodium-potassium ATPase pumps 31. Hence, we used KN92, a more selective CaMKII inhibitor, as a control, the results suggested that both KN93 and KN92 can significantly increase the CRT expression in B16F10 cells, compared with the PBS group. Moreover, the expression of CRT in KN93 group was higher than that in KN92 group** (Figure S7)**, indicating that the induction of ICD may be due to the inhibition of CAMKII and K+ channels.

As already demonstrated, the MRK hydrogel had obvious synergistic effects on growth inhibition and induction of ICD levels in B16F10 cells. Along with the direct antitumor activity of the hydrogel scaffold itself, we sought to explore whether the presence of other related mechanisms could explain the detected synergistic effects further. For this purpose, real-time confocal imaging studies and flow cytometry analysis were performed with the MR_52_-Cy5 hydrogel to study intracellular uptake. Confocal imaging results showed that the MR_52_-Cy5 group had much stronger fluorescence signals in B16F10 cells than the free Cy5 group, especially at 0.5 h, 1 h, and 2 h (**Figure 2G**). The flow cytometry analysis further revealed that Cy5 uptakes in the MR_52_-Cy5 group were 4.6-fold, 4.8-fold, 4-fold, and 2.3-fold those in the free Cy5 group at 0.5 h, 1 h, 2 h, and 4 h, respectively (**Figure 2H-I**). Melittin can insert into cell membrane and form pores 32, which may lead to an increase in intracellular uptake of KN93 encapsulated in MRK hydrogel. To further validate the effect of melittin, we examined whether R-Cy5 (not including melittin) can facilitate intracellular uptake of Cy5 by flow cytometry analysis. According to **Figure S8**, compared with the free Cy5 group, R-Cy5 did not significantly increase the uptake of Cy5, whereas, Cy5 uptakes in the MR_52_-Cy5 group were much more than other groups. In sum, these results indicate that the loading of KN93 into the MR_52_ hydrogel might increase the uptake of KN93 to enhance antitumor and ICD effects on B16F10 cells.

**Figure 3:**
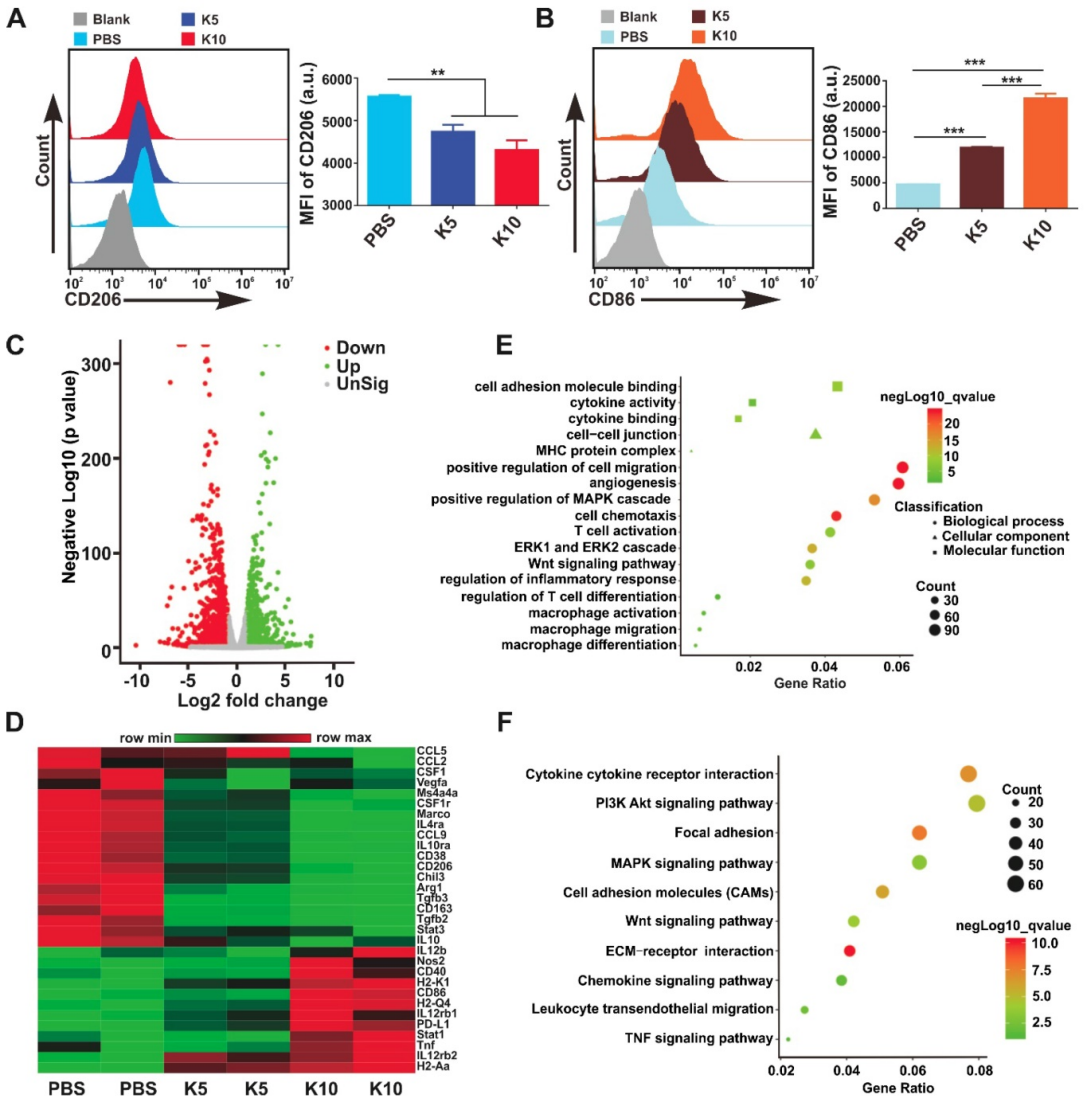
** Targeting CAMKII to reset TAMs toward antitumor phenotypes. (A, B)** Flow cytometry analyses of the expression of CD206 and CD86 in BMDMs. BMDMs were treated with KN93 (5 *μ*M or 10 *μ*M) for 24 h. Data are presented as the mean ± SEM (n = 3). **(C, D, E, F)** RNA sequencing analyses. BMDMs were cocultured with PBS, KN93 (5 *μ*M), or KN93 (10 *μ*M) for 24 h. RNA was extracted for RNA sequencing, and the sequence data were analyzed. **(C)** A volcano plot showing the up-regulated or insignificantly expressed or down-regulated genes when comparing the KN93 (10 *μ*M)-treated group with the PBS group. **(D)** Heatmaps illustrating the log_2_-fold change of M1- and M2-related gene sets, as indicated. **(E, F)** GO and KEGG enrichment analyses identifying the activation of specific canonical pathways as indicated in BMDMs treated with KN93 (10 *μ*M) or PBS.

**Targeting CAMKII to Reprogram TAMs toward the M1 Phenotype.** TAMs are an important component of TME, which are often educated into the M2 phenotype by tumor-derived cytokines, such as M-CSF, IL-4, IL-13, and IL-10. Several studies have revealed that TAMs can promote tumor immune escape, tumor growth, angiogenesis, and metastasis. Targeting TAMs to reset M2 towards the anti-tumor M1 phenotype is considered as a promising strategy in cancer immunotherapy 1, 9. Interestingly, we found in the current investigation that the expression level of CAMKII mRNA in M2 was much higher than that in M1 (**Figure S10**). Based on this finding, the CAMKII specific inhibitor KN93 was used to explore the effect on bone marrow-derived macrophages (BMDMs). Results showed that the IC50 for KN93 against BMDMs was about 80 *μ*M (**Figure S11**). Therefore, we used KN93 with various concentrations where the cell viability of BMDMs was not affected in subsequent functional tests. Strikingly, KN93 significantly increased the expression of CD86 (M1 marker) and reduced CD206 (M2 marker) expression in a concentration-dependent manner by the flow cytometry (**Figure 3A-B**). These effects were also observed both in IL-4/13-polarized M2 and IL-10-polarized M2 (**Figure S12**). To further verify whether KN93 could affect the polarization of BMDMs at the transcriptome level, we cultured BMDMs with KN93 (5 *μ*M or 10 *μ*M) or PBS for 24 h and analyzed the global transcriptional profile in cells through RNA sequencing. Compared with control cells, KN93-treated BMDMs expressed significantly higher levels of M1-related transcriptional factors, cytokines and other molecules and lower levels of M2-associated transcriptional factors, cytokines and other molecules.

For example, M1-associated Nos2, IL12b, Tnf, Stat1, CD86, and CD40 and histocompatibility complex-related molecules (H2-Aa, H2-M2, H2-Ab1, H2-Eb1, H2-K2, and H2-T22) increased significantly; M2-related CSF1r, CSF1, Arg1, CD206, CD163, Tgfb3, IL-10, IL-4ra, Vegfa, CCL2, CCL9, CCL12, Fn1, and Mrc2 and transcriptional factors, such as Irf5, Irf8, and Myc, reduced significantly in the KN93-treated group compared with those in control cells (**Figure 3C-D, Figure S13A-B**). To assess the effect of KN93 on BMDMs reprogramming more accurately, we performed Gene Ontology (GO) analysis and Kyoto Encyclopedia of Genes and Genomes (KEGG) analysis to identify the canonical signaling pathway activation in the cells based on differentially expressed genes (KN93 10 *μ*M vs Control or KN93 5 *μ*M vs Control). GO analysis showed that cytokine activity and binding, MHC protein complex, angiogenesis, ERK1 and ERK2 cascade, MAPK cascade, Wnt signaling pathway, macrophage differentiation, migration, and activation, T cell differentiation and activation were significantly enriched in the KN93 (10 *μ*M)-treated group compared with the control group (**Figure 3E**). According to the results of the KEGG analysis, PI3K-AKT and MAPK signaling pathways were significantly enriched in the KN93 (10 *μ*M)-treated group compared with the PBS group (**Figure 3F**). Similar results with GO and KEGG analysis were also observed in the KN93 (5 *μ*M)-treated group compared with control cells (**Figure S13C-D**). Related published studies have suggested that MAPK 15 and AKT 33 signaling pathways played an important role in reprogramming TAMs. We further identified the enriched PI3K-AKT, MAPK signaling pathways by western blotting. As shown in**Figure S14**, KN93 treatment significantly upregulated the expression of p-p38 but not MAPK other members, p-ERK1/2 and p-JNK; and significantly decreased the expression of p-AKT. These results suggested that the mechanism of reprogramming TAMs by targeting CAMKII may be through promoting the activation of p38 MAPK pathway and/or inhibiting AKT pathway activation. Conclusively, these results demonstrate that targeting CAMKII reprogramed M2 toward the M1 phenotype *in vitro*. To further verify the effects of the MRK hydrogel on BMDMs reprogramming, we cultured IL-4/13-M2 with MRK (MR_52_, 4.5 *μ*M; KN93, 10 *μ*M) or other control elements for 24 h and analyzed the expression of CD86 and CD206 by flow cytometry. Consistent with the results obtained in the KN93 group, MRK hydrogel significantly increased CD86 expression and reduced CD206 expression. The results indicate that MRK hydrogel retained the function of KN93 in reprogramming M2 toward the M1 phenotype (**Figure S16A-D**). Dendritic cells (DCs) are central regulators of T cell-mediated cancer immunity for the initiation and maintenance of immune responses against malignant cells 34. Moreover, as DCs and BMDMs have the same origin, as both derived from bone marrow, we explored whether the MRK hydrogel can promote the maturation of DCs *in vitro*. Compared with the PBS group, KN93 and MR or MRK hydrogels augmented the expression levels of the surface marker CD86, the activated marker of DCs, significantly (**Figure S16E-F**). Collectively, these results point to a promotion role for the MRK hydrogel on DCs' maturation.

**Figure 4:**
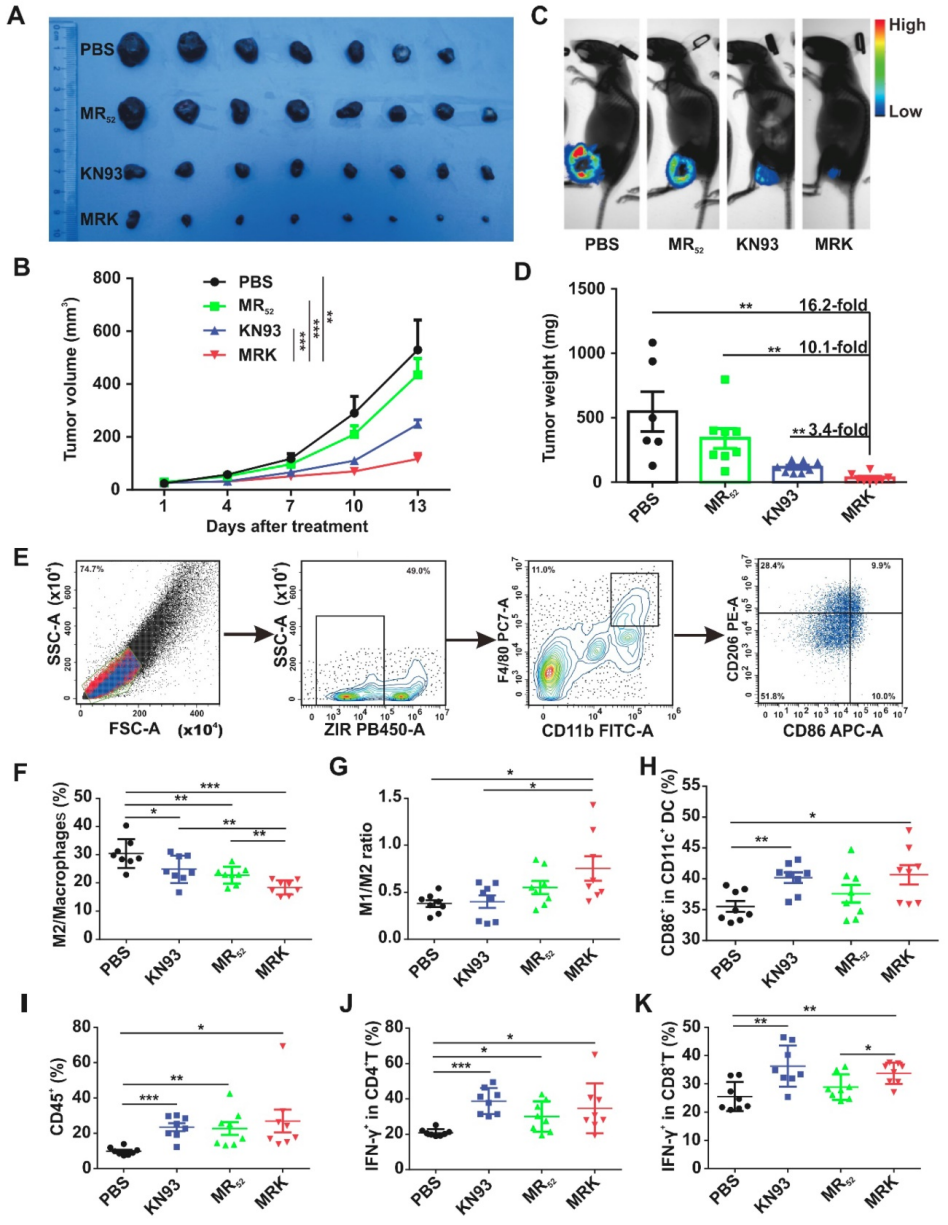
***In vivo* therapeutic effects and immune Activation of the MRK hydrogel. (A)** Photograph of dissected tumor samples in different groups as indicated. **(B)** Tumor growth curves for mice injected intratumorally with PBS, MR_52_, KN93, or MRK hydrogel. Data are presented as the mean ± SEM (n = 7-8).** (C)** Representative bioluminescence images of B16F10 tumors in C57BL/6 mice after various treatments as indicated. **(D)** Quantification of tumor weights in each group. Data are presented as the mean ± SEM (n = 7-8)**. (E)** Flow cytometry gating strategy for the detection ratio of macrophages. **(F-G)** Percentages of the M2/total macrophages **(F)** and M1/M2 ratios **(G)** within the TME of various treatment groups (M1-like TAMs: ZIR^-^CD11b^+^F4/80^+^CD86^+^CD206^-^, M2-like TAMs: ZIR^-^CD11b^+^F4/80^+^CD86^-^CD206^+^). **(H)** Percentages of activated DCs (CD11c^+^CD86^+^) within ILNs. **(I)** Ratios of CD45^+^ lymphocytes within the TME in each group. **(J)** Percentages of CD4^+^IFNγ^+^ T cells (Th1 cells) within the TME in each group. **(K)** Portions of cytotoxic CD8^+^IFNγ^+^ T cells (CTLs) within the TME in each group. Data are presented as the mean ± SEM (n = 8) for (F-K).

***In vivo* therapeutic effects and immune Activation of the MRK hydrogel.** To validate whether the proposed combination targeting immunotherapy strategy can augment antitumor effects *in vivo*, we used the MRK hydrogel to treat an established subcutaneous B16F10 mouse melanoma tumor model. In this model, B16F10 cells stably overexpressing firefly luciferase (B16-luc) were selected for subsequent bioluminescence fluorescence imaging. When the tumor volume reached about 30 mm^3^, tumor-bearing mice received a single intratumoral implant of 50 *μ*L PBS, KN93 (0.4 mg per mouse), MR (1 mg per mouse) or MRK (containing 1 mg MR and 0.4 mg KN93) hydrogel (**Figure S17A**). The MR hydrogel alone showed a slight antitumor effect, and free KN93-treated mice exhibited a significant delay in tumor growth. Strikingly, the MRK hydrogel group showed significantly augmented antitumor efficacy compared with the KN93 group, with inhibition rates of 78% and 47% calculated by tumor volume, respectively, indicating the importance of a backbone with MR_52_ for the anticancer effect of the MRK hydrogel (**Figure 4A-B**). The bioluminescence fluorescence imaging results also verified that the MRK hydrogel possessed more tumor inhibition ability than the other groups (**Figure 4C**). According to** Figure 4D**, the MRK hydrogel caused 16.2-fold, 10.1-fold, and 3.4-fold more reductions in dissected tumors weights compared with the PBS, MR, and KN93 groups, respectively. Additionally, these results were in line with the TUNEL assay and Ki67 staining, which indicated that the MRK hydrogel caused the most apoptosis and the least proliferation, respectively (**Figure S17B**). Toxic effects were also evaluated by recording mice's weight changes and performing hemanalysis, biochemical analyses, and histopathological analyses for healthy tissues (including the heart, liver, spleen, lung, and kidney). No significant weight loss was observed after mice received PBS, KN93, MR, or MRK hydrogel treatment** (Figure S17C)**. Compared with the PBS control, all drug-treated groups showed no observable changes in red and white blood cell (WBC and RBC) numbers, platelet (PLT) number, hemoglobin (HGB), and liver and renal function parameters (such as glutamic pyruvic transaminase (ALT), glutamic oxaloacetic transaminase (AST), creatinine (CR), and blood urea nitrogen (BUN)) (**Figure S18A**). Post-mortem histopathological analyses of the heart, liver, spleen, lung, and kidney indicated no appreciable abnormality or noticeable organ damage in the dose used in all drug-treated groups (**Figure S18B**).

We assessed the ability of the local delivery of a KN93-loaded MR hydrogel to trigger innate and adaptive antitumor immune responses *in vivo*. Subcutaneous B16F10 tumor-bearing mice were treated intratumorally with PBS, MR, KN93, or MRK hydrogel. Furthermore, inguinal lymph nodes' (ILNs) DCs of the drainage area, macrophages (**Figure 4E**), help T lymphocyte 1 (Th1) cells, and cytotoxic T lymphocytes (CTLs) in the tumors were analyzed by flow cytometry on day 8 after treatments. As shown in **Figure 4F-G**, MRK-treated mice had significant reductions in the portion of M2-like TAMs in tumors compared with other groups on day 8 post-treatment by flow cytometry. More strikingly, the MRK hydrogel group exhibited the highest ratio of M1-like to M2-like TAMs (M1/M2) compared to the other groups (M1-like TAMs: ZIR^-^CD11b^+^F4/80^+^CD86^+^CD206^-^, M2-like TAMs: ZIR^-^CD11b^+^F4/80^+^CD86^-^CD206^+^). We also analyzed the mean fluorescence intensity (MFI) of CD206 and MFI of CD86/ MFI of CD206 ratio in TAMs (ZIR^-^CD11b^+^F4/80^+^) from various treatment groups.

The results were in line with the portion of M2-like TAMs and ratio of M1/M2 (**Figure S19**). Furthermore, we examined possible changes of mature DCs, expression of CD11c^+^CD86^+^, in the drainage ILNs area, significantly higher in the KN93 and MRK hydrogel-treated groups than in the PBS group (**Figure 4H**). In summary, these results demonstrate that the MRK hydrogel could reprogram TAMs toward the M1 phenotype and promote DCs' maturation *in vivo*. An increasing body of evidence indicates that Th1 cells and CTLs play an important role in adaptive antitumor immune responses 35. In that regard, we compared the ratio of Th1 cells and CTLs in tumors among PBS, KN93, and MR and MRK hydrogels. The gating strategy for Th1 cells and CTLs, defined by ZIR^-^CD45^+^CD3^+^CD4^+^IFN-γ^+^ and ZIR^-^CD45^+^CD3^+^CD8^+^IFN-γ^+^ cells, respectively, is shown in **Figure S20**. According to **Figures 4I-K**, CD45^+^ lymphocytes and Th1 cells infiltrated significantly into all drug-treated groups compared with the PBS group. More importantly, the MRK hydrogel and KN93 caused significant intratumoral CTLs' infiltration compared with the PBS group. Collectively, these results suggest that the MRK hydrogel could trigger strong adaptive T cell-mediated antitumor immunity.

**Figure 5:**
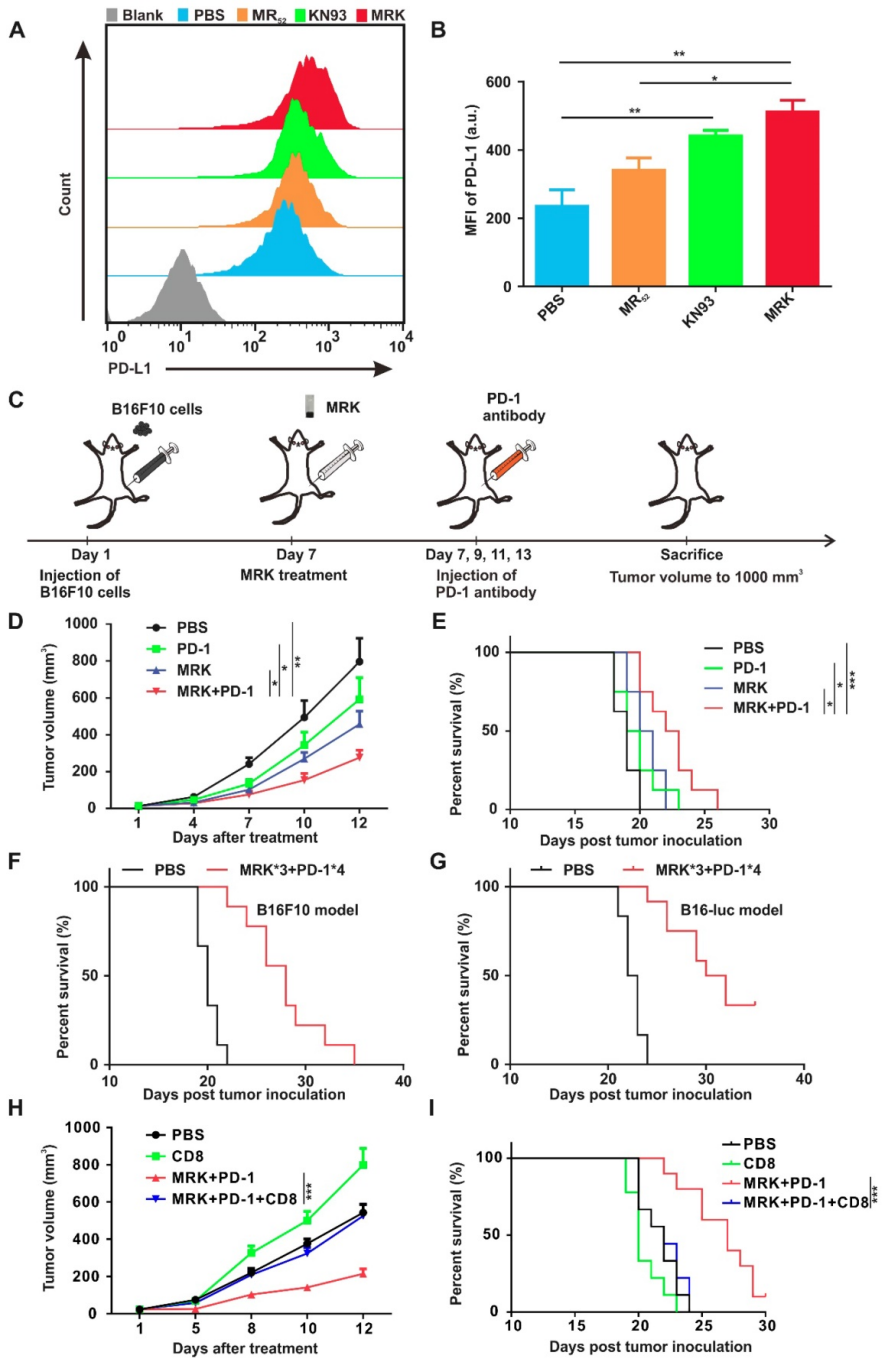
** Combining the MRK hydrogel and the PD-1 antibody exerted synergistic antitumor effects. (A, B)** Detection of PD-L1 expression levels in BMDMs after various treatments, as indicated by flow cytometry. Data are presented as the mean ± SEM (n = 4). **(C)** Schematic illustration of the combination of the MRK hydrogel and PD-1 antibody treatment schedule for subcutaneously implanted B16F10 melanoma model.** (D)** Tumor growth monitored for 12 days in mice after various treatments, as indicated. Data are presented as the mean ± SEM (n = 8). **(E)** The long-term survival in the corresponding treatment groups recorded according to the Kaplan-Meier analysis (n = 8). **(F)** Survival curves for the treated group (receiving three stitches of the MRK hydrogel and four stitches of the PD-1 antibody treatment) and PBS group (n = 9) in the B16F10 melanoma model. **(G)** Survival curves for the treated and controlled mice, as indicated (n = 12) in the B16-luc melanoma model. **(H)** Tumor growth monitored in C57BL/6 mice (n = 9-10) with B16-luc melanoma that were treated with anti-CD8 neutralizing antibody and/or MRK plus anti-PD-1 for 12 days. **(I)** Survival curves for the corresponding treatment groups according to the Kaplan-Meier analysis (n = 9-10).

**Combined MRK Hydrogel and PD-1 Antibody Exerted Synergistic Antitumor Effects.** As shown in **Figure 3D**, KN93 elevated the expression of PD-L1 mRNA in a concentration-dependent manner significantly. Hence, we demonstrated further that the expression of the PD-L1 protein on the cell membrane of BMDMs was increased extensively by KN93 treatment in a concentration-dependent manner (**Figure S21**). At the same time, MRK hydrogel-treated BMDMs displayed the highest levels of PD-L1 expression compared with PBS, KN93, or MR hydrogel treatment (**Figure 5A-B**). It is well known that PD-L1's high expression in TME could inhibit T cell activation and induce T cell apoptosis by interacting with PD-1^36^. Therefore, we speculated that combining the MRK hydrogel with a PD-1 antibody could exert synergistic therapeutic effects on tumors. To confirm this hypothesis, we used the subcutaneous B16F10 tumor-bearing mouse melanoma model. When the tumor volume reached about 30 mm^3^, tumor-bearing mice were randomly divided into four groups, treated respectively with PBS, PD-1 antibody, MRK hydrogel, and PD-1 antibody + MRK hydrogel. The treatment process is shown in **Figure 5C**. As depicted in **Figure 5D**, treatment with only the PD-1 antibody resulted in a slight tumor inhibition, which was not statistically significant compared with the result from the PBS group. In contrast, mice receiving MRK hydrogel + PD-1 antibody treatment exhibited the highest inhibitory efficacy against B16F10 tumors. The tumor sizes in mice were associated with their survival (**Figure 5E**). Mice receiving combination therapy showed the longest survival time compared to the other groups. To acquire a better treatment effect, we treated tumor-bearing (B16F10) mice with three periodical intratumoral injections of the MRK hydrogel and four periodical intraperitoneal injections of the PD-1 antibody. According to **Figure 5F**, mice in the treatment group had longer survival time noticeably. Mice in the PBS group all died 22 days after tumor inoculation. In contrast, 22% of mice in the treatment group survived more than 30 days. Fascinatingly, melanoma patients are known to have good response rates (about 30%) to PD-1 antibodies in the clinic^37^, ^38^, despite B16F10-bearing melanoma mice exhibiting a poor response to the PD-1-only antibody treatment (**Figure 5D**). Therefore, for further verification of the effects of combined therapy (MRK*3 and PD-1*4), we used the B16-luc melanoma mice model with more immunogenicity. The combination therapy conferred a survival rate of about 33% against subcutaneous melanoma mice on day 35 (**Figure 5G**). Moreover, no significant weight change was observed after the administration of the combined therapy in B16F10 and B16-luc models (**Figure S22A-B**). To further explore the roles of CD8^+^ T cells in the antitumor activity of combination treatment with MRK and anti-PD-1, we depleted CD8^+^ T cells by CD8 neutralizing antibody (**Figure S23-24**). We found CD8^+^ T depletion significantly attenuated the inhibitory effect of MRK plus PD-1 antibody on B16-luc melanoma (**Figure 5H-I**). Altogether, combined MRK hydrogel and PD-1 antibody exerted synergistic antitumor effects by a CD8^+^ T cells-dependent pathway.

**Figure 6:**
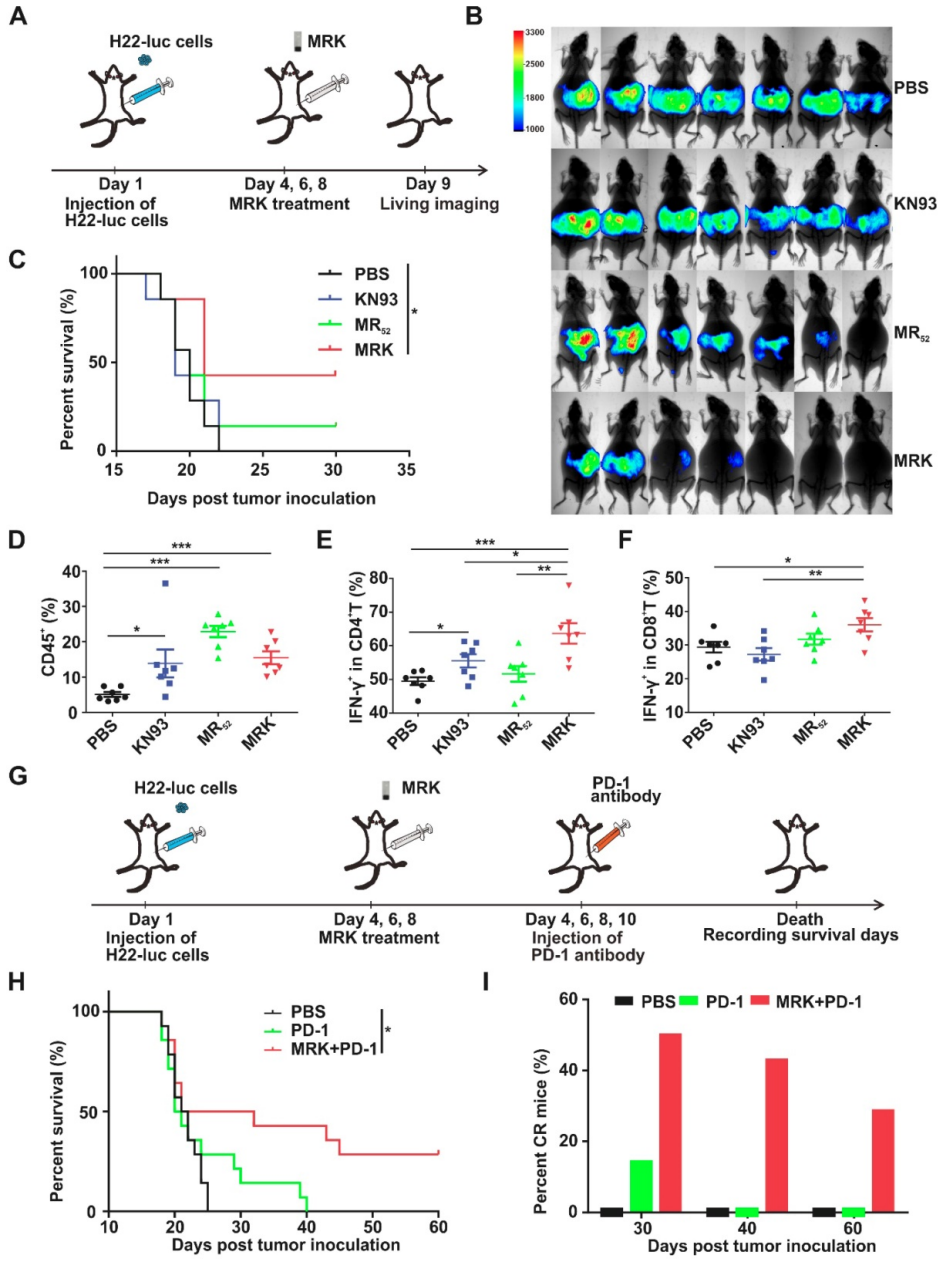
** Therapeutic effects of MRK alone and combined with the PD-1 antibody in MAs of the H22 hepatoma model. (A)** Schematic illustration of the MRK hydrogel treatment schedule for the intraperitoneally injected H22-luc hepatoma ascites model. **(B)** Bioluminescence images of H22 hepatoma ascites mice after various treatments, as indicated (n = 7).** (C)** Survival percentages in each group (n = 7).** (D)** Ratios of CD45^+^ lymphocytes within ascites in each group. **(E)** Percentages of Th1 cells within ascites in each group. **(F)** Portions of cytotoxic CTLs within ascites in each group. Data are presented as the mean ± SEM (n = 7) for (D-F). **(G)** Schematic illustration of the MRK hydrogel and PD-1 antibody treatment schedule for the H22-luc hepatoma ascites model. **(H)** Survival curves for the treated and controlled mice, as indicated (n = 14).** (I)** Percentages of CR mice in each group (n = 14).

**The Antitumor Effect of Combination Treatment in Malignant Ascites (MAs) Models.** MAs present a considerable clinical challenge to their management. When hepatoma patients develop the symptoms of ascites, it usually indicates a sign of advanced disease and poor prognosis 39, 40. Accordingly, we evaluated the effects of MRK and/or PD-1 antibody in advanced hepatoma ascites model further. H22-luc cells were injected intraperitoneally, and three days later, tumor-bearing mice were given the treatment of PBS, KN93, and MR_52_ or MRK hydrogels (**Figure 6A**). Tumor growth was monitored by the bioluminescence signals of H22-luc cells (**Figure 6B**). KN93-treated mice showed no superior tumor inhibition to mice in the PBS group. In contrast, mice receiving the MR_52_ and MRK hydrogels showed tumor inhibition effects. Strikingly, the MRK-treated mice exhibited less tumor burden than mice in the MR group. At the same time, survival analysis results suggested that the MRK group had the longest survival time compared to the other groups (**Figure 6C**).

Furthermore, on day 6, post-treatment, lymphocytes, Th1 cells, and CTLs in ascites were detected by flow cytometry. According to **Figure 6D**, all drug-treated groups exhibited significantly more amounts of infiltrating lymphocytes than the PBS group. More importantly, we observed that the MRK hydrogel treatment caused the most substantial increase in the proportions of Th1 cells and CTLs in contrast to other controls (**Figure 6E-F**), indicating that the MRK hydrogel could trigger T cell-mediated adaptive antitumor immunity.

In H22-luc model, we detected the expression of PD-L1 in living cells of ascites after KN93 treatment. Compared with the PBS group, KN93-treated group significantly increased PD-L1 expression in ascites microenvironment (Figure S25A-B). These results further confirmed the necessity of the combination therapy of anti-PD-1 and MRK. We explored whether combined MRK hydrogel with PD-1 antibody had better tumor inhibition in the H22 hepatoma ascites model than individual treatments. In this experiment, abdominal H22 tumor-bearing mice were injected intraperitoneally with PBS, PD-1 antibody, or MRK hydrogel + PD-1 antibody every one day (**Figure 6G**). According to **Figure 6H-I**, the survival curve showed that 50% of mice survived at least 30 days, 43% of mice survived at least 40 days, and 29% of mice survived at least 60 days (ablation effect) after treatment with MRK hydrogel + PD-1 antibody. In contrast, none of the mice survived in any of the control groups after 40 days. Moreover, no obvious weight loss was observed after the combined therapy in H22-luc models (**Figure S26**)**.** We also performed an experiment to evaluate the therapeutic effects of MRK combined with PD-1 antibody in MAs of the H22-luc hepatoma model directly (four groups: PBS, MRK, PD-1, MRK and PD-1). As revealed in**Figure S27**, the MRK combined with PD-1 group had the longest survival time compared to the other groups. In summary, these data suggest that the combination of MRK hydrogel and PD-1 antibody was powerful *in vivo* antitumor agent.

## Discussion

TAMs are an essential component of TME and often play a significant role in promoting tumor progression. Accordingly, simultaneously targeting tumor cells and microenvironmental TAMs is a promising strategy for cancer immunotherapy 6, 9. Several studies have verified that CAMKII plays an oncogenic role in various tumors, such as leukemia, melanoma, and hepatocellular carcinoma 16, 18. Here, we initially found that specifically inhibiting CAMKII with KN93 can reset TAMs towards the antitumor M1 phenotype. In so doing, we have designed a multidimensional therapeutic platform that consists of a simple melittin/KN93-containing peptide hydrogel (MRK hydrogel) for potent targeted immunotherapy against subcutaneous melanoma and hepatoma ascites models with extensive peritoneal metastases. The MRK hydrogel offers a melittin and KN93-based direct tumor cell-killing and induction of ICD effects, reprogramming of TAMs towards M1 macrophages, and generation of rapid innate and adaptive immune-activating responses. Moreover, we observed an up-regulation in PD-L1 in TAMs upon exposure to the MRK hydrogel. Thus, the combination of the MRK hydrogel with anti-PD-1 therapy can improve antitumor efficacy further.

Biomaterials and nanotechnology (including hydrogel) can be used to alter the colocalization, biodistribution, and release kinetics of therapeutic drugs and immunomodulatory molecules, thereby improving the efficacy and safety of these loading drugs relative to systemic administration 41-43. On the other hand, traditional hydrogel scaffolding only acts as a reservoir to control the release of loaded medications but has no direct antitumor or immunomodulatory function 25, 44, 45. Our team developed a melittin-RADA_32_ hydrogel (MR_60_) in previous studies and had shown in a prior investigation that an MR_60_ hydrogel, consisting of melittin and RADA_32_, can directly kill tumor cells and induce DCs maturation. We have also demonstrated that the RADA_32_-melittin fusion peptide can reduce the hemolysis effect of melittin significantly by forming cross-linked nanofibers 29, 46. However, RADA_32_ as a building block for MR_60_ was relatively long, thus raising difficulties and costs in synthesis. To find a more suitable melittin-encapsulated hydrogel scaffold, we compared a series of melittin-(RADA)_n_ (n=4, 6, 8) hybrid peptide sequences. We confirmed that melittin-(RADA)_6_ peptide outcompeted in terms of gel-formation ability and *in vitro* antitumor activity. Our results suggest that MR_52_ had a gel-formation ability similar to that of MR_60_, but MR_52_ had a better killing effect against B16F10 than MR_60_ (**Figure S1**)_._ Therefore, we chose the MR_52_ hydrogel scaffold to load KN93, creating an MRK hydrogel. In summary, we have designed an excellent melittin-encapsulated hydrogel scaffold with optimal characteristics in this study.

MAs present a considerable challenge to the management of various tumors, mainly including hepatoma, ovarian cancer, and gastric cancer in clinical settings. MAs are nearly incurable with the current standard therapy and respond poorly to systemic chemotherapy. Although intraperitoneal chemotherapy has some effects in the control of MAs, relapse and drug resistance remain significant challenges. Moreover, patients receiving systemic or intraperitoneal chemotherapy also suffer from more pain and fatigue, as well as higher hematological, gastrointestinal, and metabolic toxicities 40. Notably, in mouse models of MAs, few studies have found some efficient strategies that only prolong the survival time to some extent; disappointedly, mice with MAs are seldom cured in these studies 47, 48. Here, we designed a combination treatment strategy based on the MRK hydrogel and PD-1 antibody against hepatoma ascites with extensive peritoneal metastases. Remarkably, the combined MRK hydrogel and PD-1 antibody therapy achieved a cure rate of approximately 30% for hepatoma ascites with widespread peritoneal metastases. The combination therapy also conferred a cure rate of about 33% against subcutaneous melanoma mice.

Using the MRK hydrogel for cancer management may offer the following advantages: (1) localized sustained KN93 delivery. In theory, controlled localized drug delivery could enhance therapeutic efficacy. We observed that the Cy5 release rate from the MR_52_-Cy5 hydrogel in the subcutaneous model decreased significantly. (2) The direct tumor cell-killing effects of MRK. CAMKII plays an oncogenic role in various tumors, and the use of the special inhibitor KN93 can inhibit tumor growth; based on the killing effect of melittin, the MRK hydrogel can exert a synergistic tumor inhibition effect. (3) Increasing the uptake of KN93. As our results show, the MR_52_-Cy5 hydrogel increased the uptake of Cy5 in B16F10 melanoma cells using confocal imaging assay and flow cytometry, based mainly on the function of melittin. Accordingly, the MRK hydrogel could enhance the uptake of KN93 for tumor cells to promote antitumor effects. (4) Immune-stimulating ability. On the one hand, the MRK hydrogel can cause ICD in tumor cells, which can exert the activation of draining lymph nodes DCs *in vivo* further; On the other hand, the KN93 loaded in the MRK hydrogel can reprogram TAMs towards anti-tumor M1 macrophages, and MR_52_ and KN93 can also promote DCs' maturation. (5) Combination with a PD-1 antibody. The MRK hydrogel can increase the expression level of PD-L1 in TAMs, which provides a good rationale for combination therapy involving the MRK hydrogel and a PD-1 antibody. This combination strategy produced a cure rate of up to about 30% when used for hepatoma MAs treatment. Additionally, it showed potent therapeutic efficacy in a subcutaneous B16-luc melanoma model. (6) Ease of synthesis and low synthesis costs. We shortened the length of RADA and screened out MR_52_ further, which has lower synthesis costs and better antitumor effects than MR_60_. (7) Excellent biocompatibility and biodegradability. The MR_52_ hydrogel is composed of saline and peptides, which can be biodegraded in the presence of proteinase K. In summary, we have developed a combined targeted immunotherapy strategy based on the controlled release of targeted drug KN93 from the MRK hydrogel scaffold and PD-1 antibody. In the future, the MRK hydrogel may serve as a prospective platform for antitumor treatment.

## Conclusion

A promising cancer immunotherapy strategy requires not only the killing effects on tumor cells but also the regulation of TAMs reprogramming and differentiation into M1-TAMs. In summary, we have created an excellent KN93-encapsulated melittin hydrogel (MRK) with optimal characteristics in this study. The MRK hydrogel can provide a synergistic tumor suppression effect against melanoma and MAs through reshaping TMEs. Due to its localized sustained KN93 delivery, direct tumor cell-killing effects and improved uptake of KN93 and immune-stimulating ability, the MRK hydrogel elicits strong antitumor immune responses and offers potent therapeutic efficacy for melanoma and MAs. The MRK hydrogel could be further combined with PD-1 antibody to achieve a cure rate of approximately 30% for MAs with widespread peritoneal metastases.

## Supplementary Material

Supplementary experimental section and figures.
