# Supplementary material for: Targeting CAMKII to reprogram tumor-associated macrophages and inhibit tumor cells for cancer immunotherapy with an injectable hybrid peptide hydrogel
Source: Theranostics. 2020 Feb 10;10(7):3049–63. doi: 10.7150/thno.42385 (PMC7053188; doi:10.7150/thno.42385)
Supplement: Supplementary file 1 — Supplementary experimental section and figures. [file thnov10p3049s1.pdf]

Targeting CAMKII to reprogram tumor-associated macrophages and inhibit tumor cells for cancer immunotherapy with an injectable hybrid peptide hydrogel

Xiaomeng Dai[†1], Jingshu Meng[†1], Suke Deng[†1], Lingling Zhang[†], Chao Wan[†], Lisen Lu[†], Jing Huang[†], Yan Hu[†], Zhanjie Zhang[†], Yan Li[†], Jonathan F. Lovell[‡], Gang Wu[†], Kunyu Yang[†], and Honglin Jin[†*]*

[†]Cancer Center, Union Hospital, Tongji Medical College, Huazhong University of Science and Technology, Wuhan 430022, China

[‡]Department of Chemical and Biological Engineering, University at Buffalo, State University of New York. Buffalo, New York 14260, USA

[1] These authors contributed equally to this work.

[*]Correspondence: Cancer Center, Union Hospital, Tongji Medical College, Huazhong University of Science and Technology, Wuhan 430022, China. Fax: +86-27-65650733; Tel: +86-27-85873100. Email: Kunyu Yang, yangkunyu@medmail.com.cn; Honglin Jin, jin@hust.edu.cn

Keywords. Hydrogel, cancer immunotherapy, tumor-associated macrophages, anti-PD-1,

CAMKII.

Experimental Section

Materials. KN93 Phosphate (KN93 H3PO4) and KN-92 phosphate (KN92 H3PO4) were purchased from Selleck. $RADA_{16}$-melittin fusion peptide (Ac-RADARADARADARADA-GG-GIGAVLKVLTTGLPALISWIKRKRQQ-NH2), $RADA_{24}$-melittin fusion peptide (Ac-RADARADARADARADARADARADA-GG-GIGAVLKVLTTGLPALISWIKRKRQQ-NH2), $RADA_{32}$-melittin fusion peptide (Ac-RADARADARADARADARADARADARADARADA-GG-GIGAVLKVLTTGLPALISWIKRKRQQ-NH2), and $RADA_{24}$ peptide (Ac-RADARADARADARADARADARADA-NH2) were synthesized by Bankpeptide Ltd. (Hefei, China).

Hydrogel Synthesis. For the construction of the MR hydrogel, 10 mg MR peptides were dissolved entirely in 1 mL of 0.9% NaCl solution and kept at 4°C overnight to form a stable gel. The MRK hydrogel was obtained in the same way by dissolving 10 mg MR peptides in 1 mL of different concentrations of the KN93 solution (which was already prepared by dissolving KN93 powder in 0.9% NaCl solution). The MR_{52}-Cy5 was obtained by dissolving 10 mg MR_{52} peptides in 1 mL of Cy5 solution (which was already prepared by dissolving different amounts of Cy5 powder (A8108, APExBIO) in 0.9% NaCl solution). All processes were carried out under sterile conditions. The R-Cy5 ($RADA_{24}$-Cy5) was also obtained in same manner.

Mice and Animal Experiment. Female C57BL/6 and Male BALB/c (6-week-old) were purchased from HBCDC (Wuhan, China). All mice were kept in accordance with protocols that had been approved by the Hubei Provincial Animal Care and Use Committee and guidelines of the Animal Experimentation Ethics Committee of the Huazhong University of Science and

Technology (HUST, Wuhan, China). In the B16 melanoma subcutaneous model, 5×10^5 cells were injected subcutaneously into the right flank of female C57BL/6. Seven days later, the mice were divided randomly into different groups and treated with intratumoral administrations of 1× phosphate-buffered saline (PBS, 50 μL), KN93 (0.4 mg per mouse, 50 μL), MR (1 mg per mouse, 50 μL), or MRK (containing 1 mg MR and 0.4 mg KN93, 50 μL). In the H22 hepatocarcinoma ascites model, 3×10^5 cells were implanted intraperitoneally (i.p.) into male BALB/c mice. Three days later, the mice were divided randomly into different groups and treated with intraperitoneal injections of PBS (100 μL), KN93 (0.08 mg per mouse, 100 μL), MR (1 mg per mouse, 100 μL), or MRK (containing 1 mg MR and 0.08 mg KN93, 100 μL) every other day, for three times. Subcutaneous tumor sizes were measured every three days using vernier calipers and recorded based on the length (*L*) and the width (*W*) of tumors. The tumor size (*V*) was calculated per the formula $V=(L\times W^2)/2$.

$CD8^+$ T cell depletion. 200 μg/mouse of rat anti-CD8a (BioXcell, BE0061, clone 2.43) neutralizing antibodies were diluted to 125 μL in 0.9% NaCl solution and intraperitoneally injected every 4 days.

Cell Culture. The murine melanoma cell line B16F10 was generously provided by Professor Zhihong Zhang (HUST, Wuhan, China), while the murine hepatocarcinoma cell line H22 was purchased from the China Center for Type Culture Collection (Wuhan, China). Luciferase stably transfected cell lines (B16-luc and H22-luc) were established for *in vivo* experiments. Cells were cultured in an RPMI 1640 medium (Gibco) supplemented with 10% fetal bovine serum (FBS) (Gibco) at 37 °C in an atmosphere of 5% CO_2.

Cell Viability Assay. B16F10 cells were plated in 96-well-plates (5000 cells per well) and

allowed to grow for 24 h. The culture medium was then replaced by 100 μL fresh medium containing PBS, KN93 (10 μM), MR (4.5 μM), or MRK (containing 4.5 μM MR and 10 μM KN93). After 24 h of incubation, cell viability was determined using a CCK-8 assay kit (BS350B, Biosharp).

Cell Cycle Assay. B16F10 cells were seeded in 6-well-plates (3×10^5 cells per well) and allowed to grow for 24 h. Cells were then treated with PBS, KN93 (10 μM), MR (4.5 μM), or MRK (containing 4.5 μM MR and 10 μM KN93). After 24 h of incubation, cells were collected and fixed with 70% ethanol overnight at -20 °C. The next day, the cells were washed with PBS, RNA enzyme was added, and the reaction left to incubate at 37 °C for 30 min. PI was used to stain DNA content, and the cell cycle was determined using flow cytometry.

Apoptosis Assay. B16F10 cells were seeded in 6-well-plates (5×10^5 cells per well) and allowed to grow for 24 h. Cells were then treated with PBS, KN93, MR, or MRK at various indicated concentrations. After 24 h of incubation, cells were collected by trypsin digestion without EDTA, labeled with an Annexin V-FITC Cell Apoptosis Detection Kit (Biobox) according to the protocol, and then detected by flow cytometry.

Real-time Quantitative PCR. Total RNA was extracted with MicroElute Total RNA Kit R6831-01 (OMEGA) and reversed-transcribed into cDNA using HiScript III RT SuperMix (+ gDNA wiper) (Vazyme). The cDNA was amplificated utilizing the AceQ® Universal SYBR qPCR Master Mix (Vazyme) on a StepOnePlus Real-Time PCR System (Thermo Fisher Scientific). Gene mRNA levels were normalized by GAPDH. The primer sequences are shown as follows: CAMKIIα (TATCCGCATCACTCAGTACCTG, GAAGTGGACGATCTGCCATTT), CAMKIIβ (GCACGTCATTGGCGAGGAT,

ACGGGTCTCTTCGGACTGG), CAMKIIδ (TCAAGGCCGGAGCTTACGA, GAGGCTGTGATACGTTTGGCA), and CAMKIIγ (CAGATCCTGGAGAGTGTTAACC, CACAGGTTTTCCATAGGGATCT). All primers were synthesized by Sangon Biotech (Shanghai) Co., Ltd.

Western blotting. BMDMs were lysed with RIPA buffer with protease inhibitors and phosphatase inhibitor cocktails II and III on ice for 30 min. Next, the cell lysate was centrifuged at 14,000 g for 30 min. The harvested Proteins were separated by 8% SDS-polyacrylamide gel and then transferred to a PVDF membrane. After blocking with 5% skim milk powder dissolved in Tris-buffered saline containing 5% Tween-20 (TBST) for 1 h at room temperature, then incubated overnight at 4 °C with specific anti-p-p38 MAPK (4511, 1:1000), anti-p-JNK (4668, 1:1000), anti-p-Erk1/2 (4370, 1:1000), anti-p-AKT (4060, 1:2000), from Cell Signaling Technology, and anti-β-actin (GB11001, 1:1000, Servicebio, Wuhan, China). Then, the membranes were washed and incubated with secondary antibody at room temperature for 1 h. Next the membranes were washed by TBST and then visualized by NcmECL Ultra (P10100, NCM Biotech).

MR_{52}-Cy5 Hydrogel Degradation and Loading Cy5 Release *in vitro*. *In vitro* hydrogel degradation and loading cargo release were performed by adding 0.5 mL MR_{52}-Cy5 hydrogel (containing 5 mg MR and 0.05 mg Cy5 in 0.9% NaCl solution) to the bottom of a 1.5 mL Eppendorf tube, and 0.5 mL 0.9% NaCl buffer with or without 5 unit/mL proteinase K was added at 37 °C. The top buffer was replaced by fresh 0.9% NaCl at the indicated time points and collected to detect the concentration of Cy5 by using a fluorospectrophotometer (F97XP15007; Shanghai Lengguang Technology Co., Ltd., China) at an excitation wavelength of 646 nm and an emission wavelength of 662 nm. The remaining mass was accurately weighed at the indicated

time points.

KN93 release from MRK hydrogel *in vitro* by using HPLC method. KN93 release from MRK was performed by adding 0.5 mL MRK hydrogel (containing 5mg MR and 5 mg KN93 Phosphate in 0.9% NaCl solution) to the bottom of a 1.5 mL Eppendorf tube, and 0.5 mL 0.9% NaCl buffer with or without 5 unit/mL proteinase K was added at 37 °C. The top buffer was replaced by fresh 0.9% NaCl at the indicated time points. The collected supernatants were extracted by adding acetonitrile (acetonitrile:supernatants, 8:1) by centrifuging (14000 g, 10 min). then, the supernatants were filtered (0.2 μm filters) for HPLC (1100 LC/MSD Trap, Agilent, USA). A Agilent Zorbax Exlipse Plus C18 (100x4.6 mm, 3.5 μm particle size) HPLC packed column was used as the chromatographic column. The mobile phase: CH3OH 0.5% TFA/H2O 0.5% TFA (1:1, v/v), flow rate 1.0 mL/min, and detection wavelength 254 nm.

ATP Release Assays *In Vitro*. For the measurement of extracellular ATP levels, B16F10 cells were seeded in 24-well plates and given various treatments to be detected after 24 h. Cell culture supernatants in each experimental group were collected, and ATP concentrations were measured using luciferin-based ENLITEN® ATP Assay (Promega) kits in accordance with the manufacturer's protocol.

Cell Immunofluorescence and Confocal Imaging. For immunofluorescence staining, cells were seeded in 12-well cell culture dishes with 20 mm round glass coverslips for 24 h. The cells were then fixed with 4% paraformaldehyde (G1101, Servicebio) at room temperature for 15 min and permeabilized with 0.2% Triton X-100 in PBS for 15 min. After blocking with 5% BSA in PBS for 30 min, cells were incubated with Calreticulin (ab92516, Abcam, 1:500) in PBS overnight at 4 °C. Following the overnight incubation, cells were washed and stained with a

FITC secondary antibody for 1 h at room temperature, and Pholloidine (G1041, Servicebio, 1:200) was used to stain cell structures, while DAPI (G1012, Servicebio, 1:200) was used to stain nuclei. Cell immunofluorescence was observed using laser confocal microscopy (Leica TCS SP8).

Cellular Uptake Assay. For the assessment of MR-induced cellular uptake ability, B16F10 cells were seeded in 6-well plates and incubated with MR (2 μM) and Cy5 NHS ester (A8108, APExBIO, 10mg/mL, 1:20000) for 30 min, 1 h, 2 h, and 4 h, after which their uptake abilities were determined by flow cytometry.

TEM Imaging of Hydrogel. For transmission electron microscopy (TEM) imaging of hydrogels, MR and MRK hydrogels (10 mg/mL) were diluted with ultrapure water (1: 100). Total sample volumes of 10 μL were deposited on the surface of fresh copper grids for 2 min and then removed, and the grids were stained negatively with 10 μL phosphotungstic acid (5%) for 30 s. After air-drying, the grids were imaged using TEM (Titan G2 60–300, FEI Company, OR, USA).

Immunohistochemical Staining. Tumor tissues were fixed with 4% paraformaldehyde and sent to Biossci biotechnology Co. Ltd for paraffin-embedding of sections. After deparaffinization, antigen unmasking, and blocking, slides were incubated with a mouse antibody against Ki67 (ab16667, Abcam, 1:5000) overnight at 4 °C, followed by 1 h incubation at room temperature with a secondary rabbit anti-mouse immunoglobulin G (IgG) conjugated to horseradish peroxidase (CST). Slides were then washed with PBS three times, stained with 3,3′-diaminobenzidine, and counterstained with hematoxylin. TUNEL staining was performed according to instructions provided in the In Situ Cell Death Detection Kit manual, POD (Roche).

Generation and Activation of Mouse Bone Marrow-derived DCs and Macrophages. For the generation of BMDMs, bone marrow cells were isolated from femurs of male mice and cultured in an RPMI 1640 complete medium containing 20 ng/mL M-CSF (315-02-2) for 7 days. On day 7, 20 ng/mL of IL-4 (214-14-20) and 20 ng/mL of IL-13 (210-13-10) or 20 ng/mL of IL-10 (210-10-10) were added to stimulate M0 macrophages for 24 h to generate M2 macrophages **(Figure S9)**. For the generation of DCs, bone marrow cells were cultured continuously in 20 ng/mL GM-CSF (315-03-20) for 7 days (**Figure S15)**. On days 3 and 5, the culture medium was replaced, and the non-adherent cells were collected for further experiments. All stimulating factors were purchased from PeproTech.

The Gain of Tumor-infiltrating Immunocytes. Mice tumor tissues were collected, cut into pieces in PBS. After centrifugation, enzyme digestion was performed with 0.32 mg/mL collagenase V (Biosharp) and 0.5 mg/mL hyaluronidase (Biosharp) for 1 h. Tissue homogenate was made by grinding the digested tissues and adding RBC lysis buffer (Biosharp) for 15 min. Cells were then washed with PBS and collected for further experiments. To collect immunocytes in tumor ascites, ascites were centrifuged, and an RBC lysis buffer was added directly.

Flow Cytometry. For surface-stained analysis, cells were stained with anti-mouse Zombie Violet™ Fixable Viability Kit (423114), Zombie NIR™ Fixable Viability Kit (423106), CD45 (103114), CD11b (101205), CD86 (105012), CD11c (117306), CD3e (100306), CD4 (100408), CD8a (100752), and PD-L1 (124311, 124308) in recommended antibody concentrations and incubated at 4 °C for 30min. For T-cell intracellular IFN-γ (505808) cytokine staining, cells were fixed and permeabilized after stimulation with Phorbol 12-myristate 13-acetate (PMA) (ab120297, Abcam, 100 ng/mL), Monensin sodium salt (ab120499, Abcam, 1 ug/mL), and Ionomycin calcium salt (5608212, PeproTech, 100 ng/mL) for 6 h. For CD206 (141706)

staining, cells were fixed and permeabilized without stimulation. All flow cytometry antibodies were purchased from Biolegend.

RNA Sequencing. Total RNA was extracted from mice BMDMs treated with PBS or KN93 at different concentrations using a TRIzol reagent (Vazyme). Total RNA samples were sent to Bioacme Biological Technology Corporation (Wuhan, China) for further sequencing. Purified RNA and the chain-specific cDNA library were prepared after detecting qualified samples. The prepared library was checked and then sequenced on the computer. High throughput sequencing was performed on Illumina HiSeqTM2000/ Miseq platform. The primary data analyses included gene expression level analysis, gene differential expression analysis, GO enrichment analysis, and KEGG enrichment analysis. We have uploaded the RNA sequencing data to GEO repository. The GEO accession number of the sequencing data is **GSE143552.**

Bioluminescent Imaging. 1% pentobarbital sodium was used for mice anesthesia before imaging by the Bruker *In Vivo* MS FX PRO Imager. For *in vivo* luciferase images, mice were injected intraperitoneally with 150 mg/kg firefly luciferin (103404-75-7, Thermo Life). 15 min later, luminescence images were acquired with 3 min exposure time, and X-ray photographs were taken with 30 s exposure time. For the *in vivo* Cy5-dye release experiment, Cy5 or MR_{52}-Cy5 were injected subcutaneously into the right flank of female C57BL/6 and imaged (ex: 650 nm, em: 700 nm) at different time points.

Statistical Analysis

Statistical significance was analyzed using unpaired two-tailed Student's t-test, and survival rates were evaluated with the Kaplan-Meier test using the Graphpad software. Experimental results are presented as the mean ± SEM. Significant differences between the groups are indicated by **P*

<0.05, **P < 0.01, ***P < 0.001.

Supplementary figures

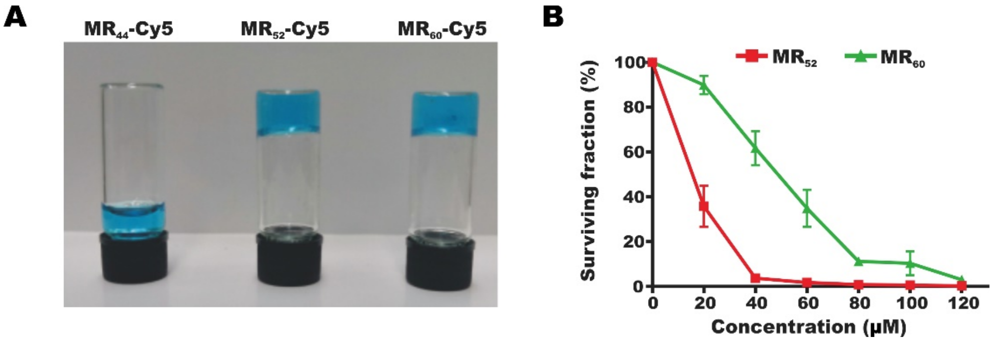

Figure S1. Screening out MR_{52} hydrogel scaffold. (A) Photographs of MR_{44}, MR_{52}, and MR_{60} hydrogels loaded with Cy5. **(B)** Cell viability examined using the CCK-8 assay.

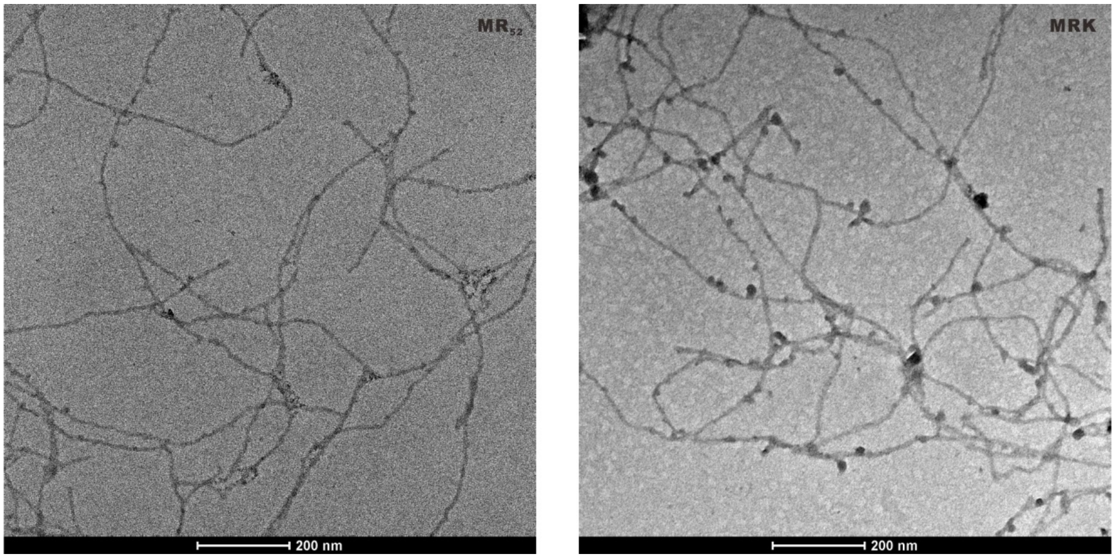

Figure S2. Representative TEM images of MR_{52} and MRK hydrogel. Scale bar, 200 nm.

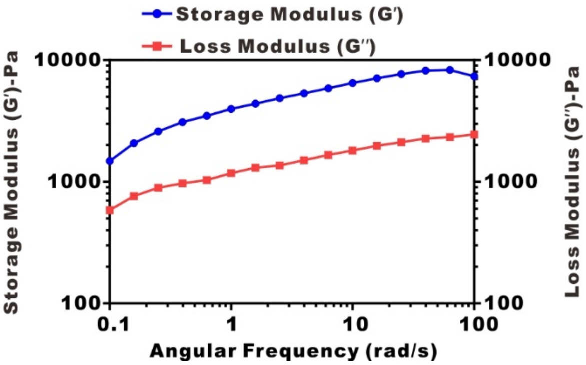

Figure S3. Frequency sweep rheological analysis of the MRK hydrogel at different angular frequency (rad/s) as indicated. Measurements were performed at a constant strain of 0.1%.

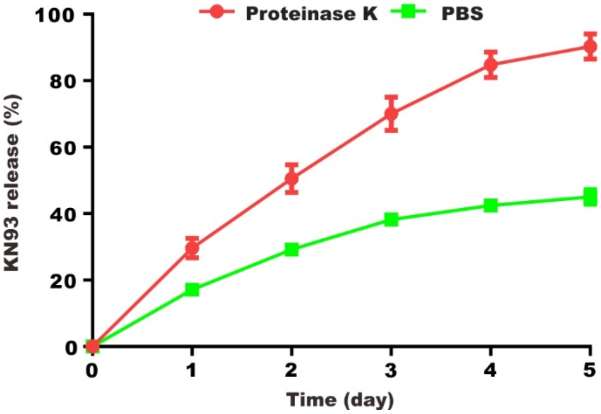

Figure S4. The KN93 release from the MR_{52} hydrogel *in vitro* in the presence or absence of proteinase K by using HPLC method

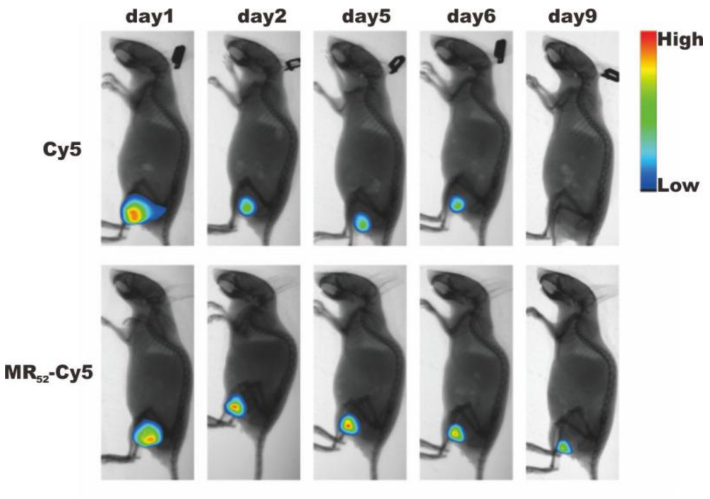

Figure S5. The NIR fluorescence imaging results of the distribution of MR_{52}-Cy5 hydrogel and free Cy5 dye after subcutaneous implantation at day 1, 2, 5, 6, and 9 *in vivo*.

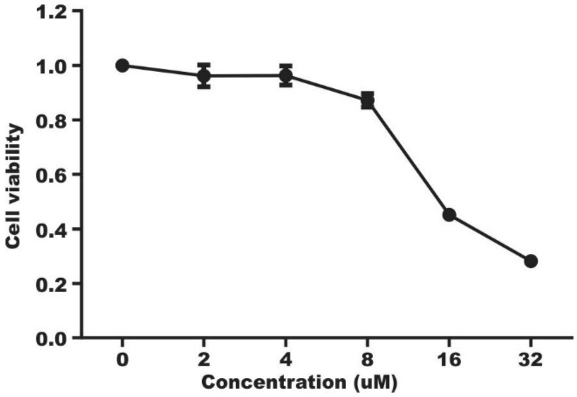

Figure S6. Cell viability assessed by CCK-8 assay at 24 h culture of B16F10 treated with indicated concentration of KN93.

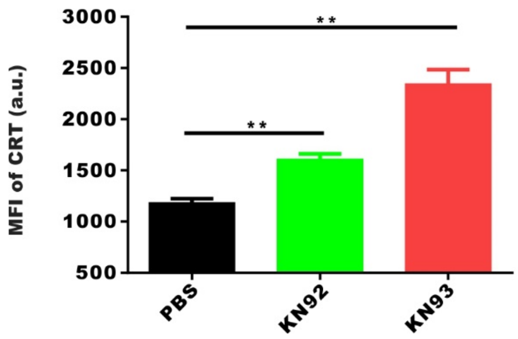

Figure S7. Flow cytometry analyses of CRT expression on the cell membrane surface after KN92 (10 uM), KN93 (10 uM), or PBS treatments for 24h. Data are presented as the mean ± SEM (n = 3)

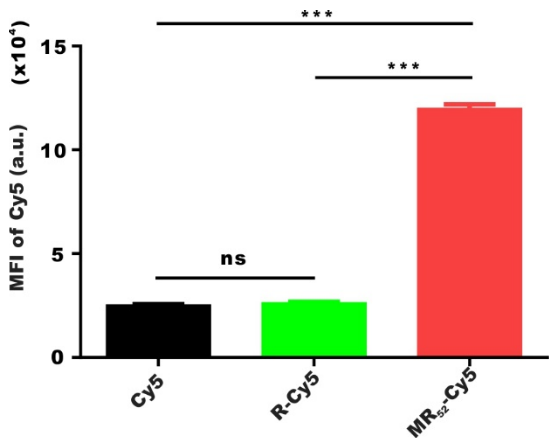

Figure S8. Flow cytometry analyses of Cy5 uptake in B16F10 cells after free Cy5, R-Cy5, or MR_{52}-Cy5 treatments at 1.5h. Data are presented as the mean ± SEM (n = 4)

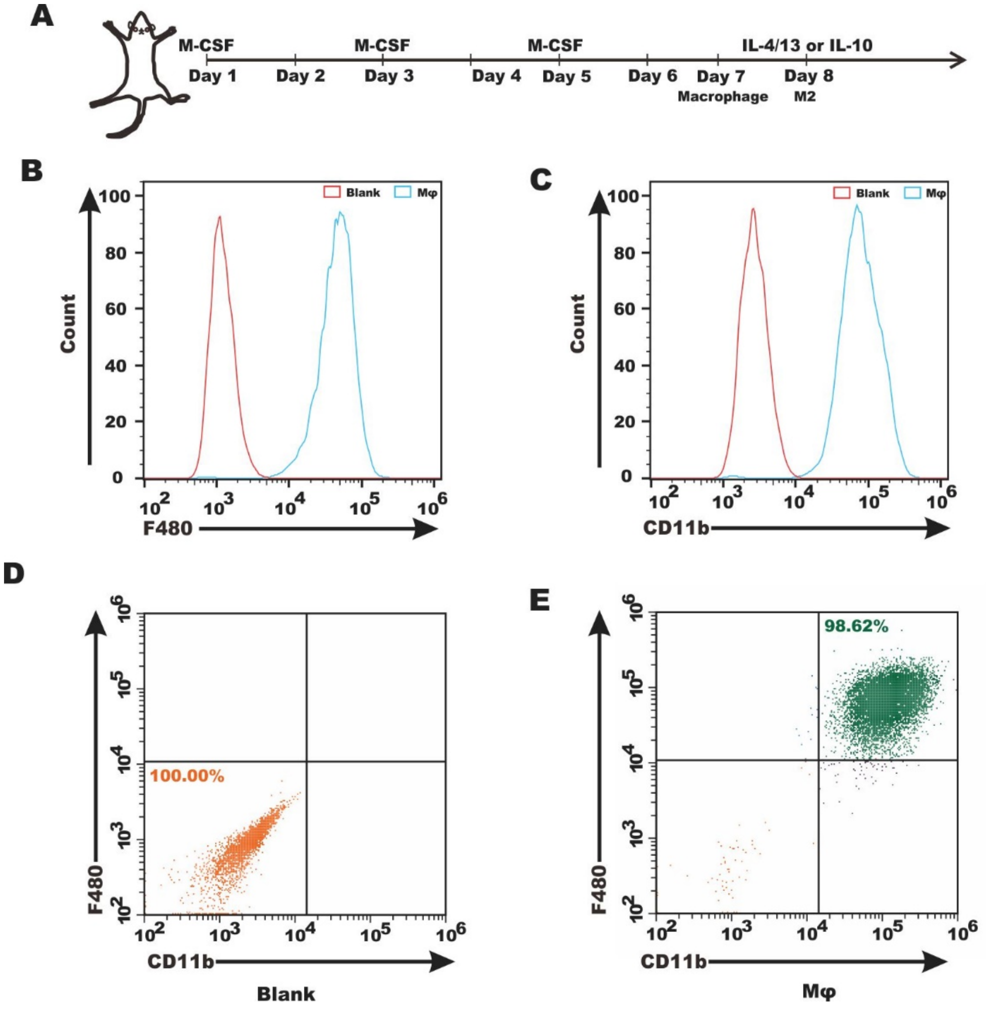

Figure S9. Induction and validation of BMDMs. (A) *In vitro* macrophage differentiation scheme of bone marrow cells cultured for 7 days with M-CSF added every two days to generate macrophages, and further polarized towards M2 by adding IL-4 and IL-13, or IL10 at the day 7. **(B-E)** Flow cytometry validating the ration of macrophage from induced bone marrow cells by M-CSF.

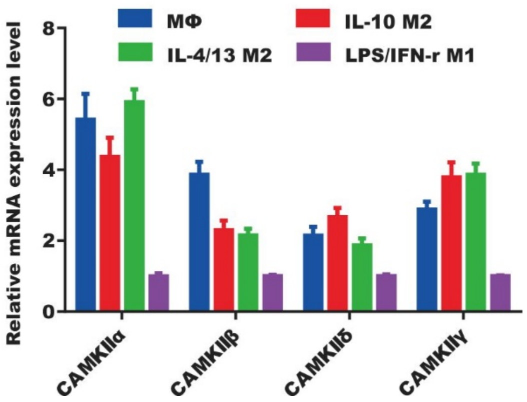

Figure S10. The mRNA expression of CAMKIIα, CAMKIIβ, CAMKIIδ, and CAMKIIγ in MΦ, IL-10 M2, IL4/13 M2, and LPS/IFN-γ M1.

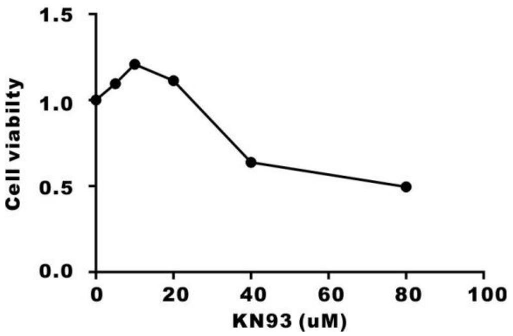

Figure S11. Viability by CCK-8 assay in 24 h culture of BMDMs in the presence of indicated concentration of KN93.

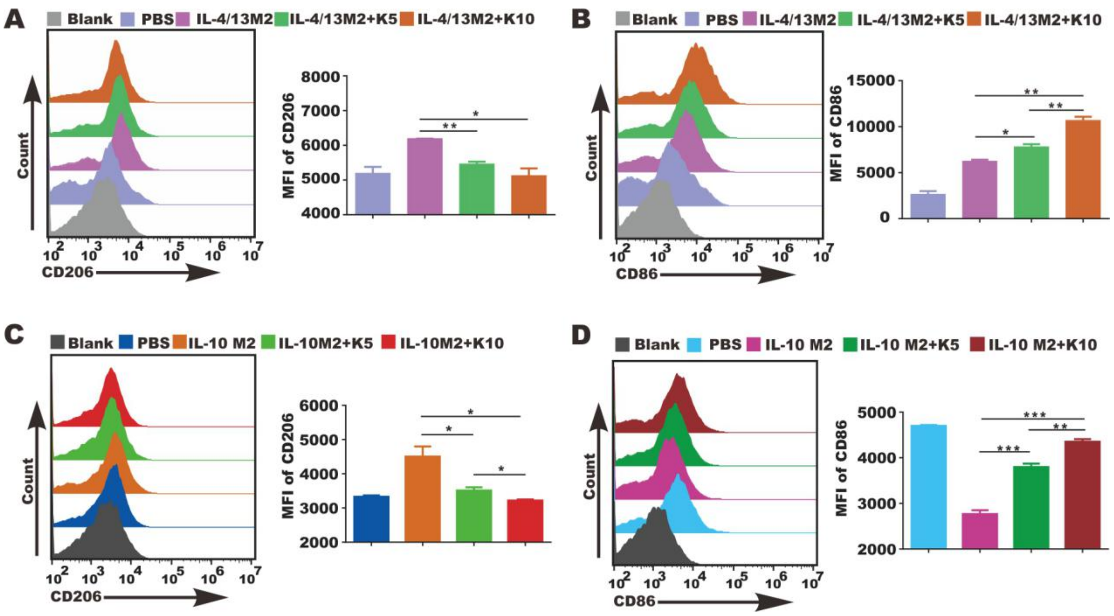

Figure S12. Blocking CAMKII to reprogram M2 toward M1 phenotype. (A-D) IL-4 and IL-13 polarized BMDMs (IL-4/13 M2) and IL-10 polarized BMDMs (IL-10 M2) were treated CAMKII special inhibitor KN93 (5 μM or 10 μM) for 24 h. The expression of CD206 and CD86 was measured by flow cytometry. Data are means ± SEM (n = 3).

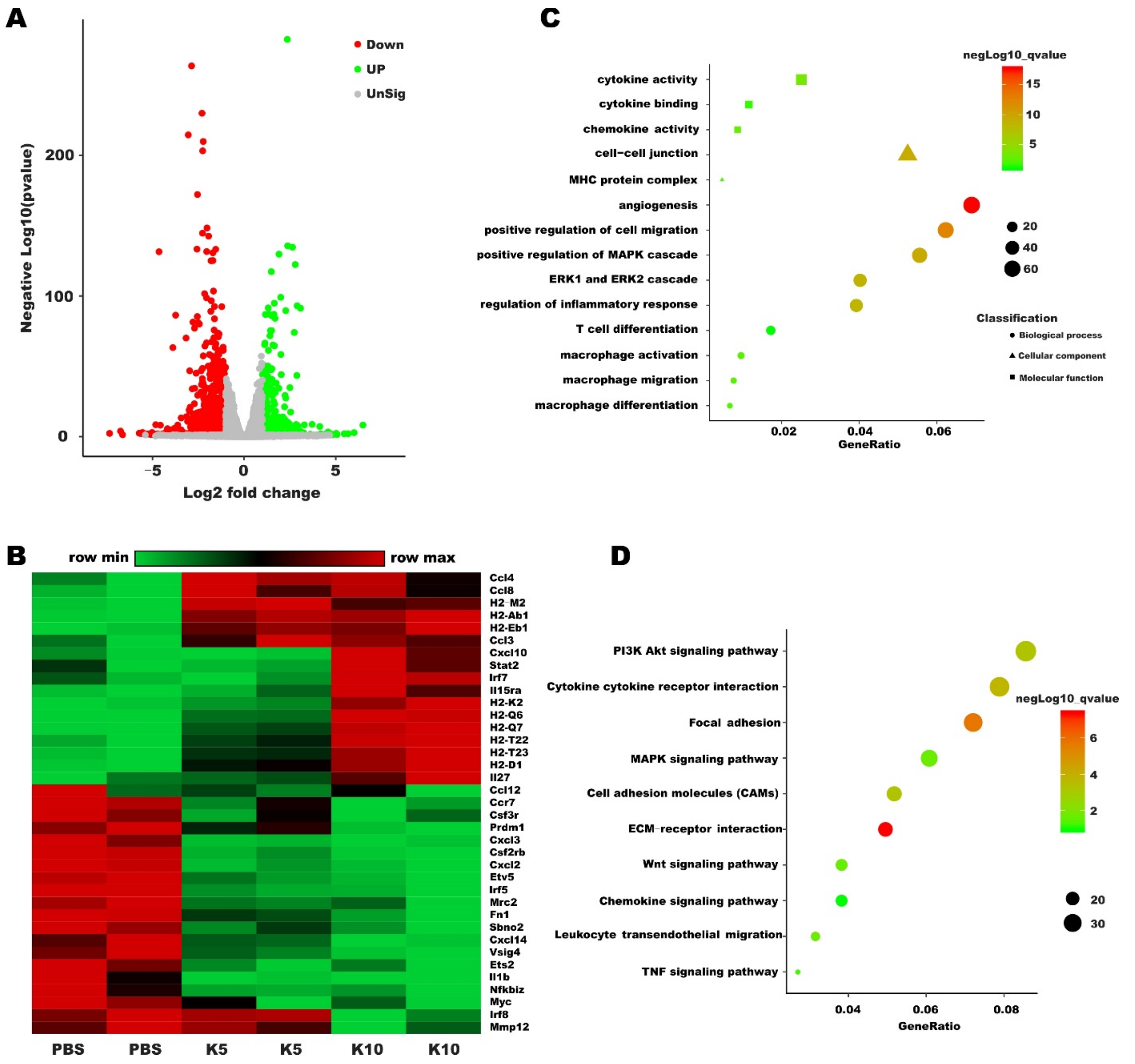

Figure S13. RNA sequencing revealed that blocking CAMKII to reset TAMs toward M1 phenotype. (A) Volcano plot showed the up-regulated, unsignificantly expressing, and down-regulated genes compared KN93 (5 μM) treating group with PBS group. **(B)** Heat maps illustrated the $\log_2$-fold change of M1 and M2 macrophages-related gene sets as indicated. **(C, D)** GO and KEGG enrich analysis identifying activation of specific canonical pathways as indicated, in BMDMs treated with KN93 (5 μM) or PBS.

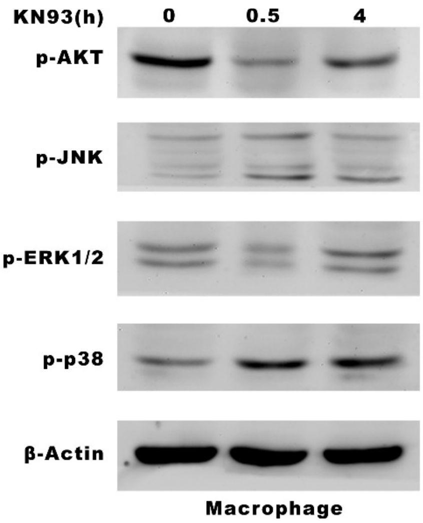

Figure S14. Western blot showing the expression of p-AKT, p-ERK1/2, p-JNK, and p-p38 in murine BMDMs cultured with IL-4 and IL-13 in the presence of 10 uM KN93 at various indicated time points.

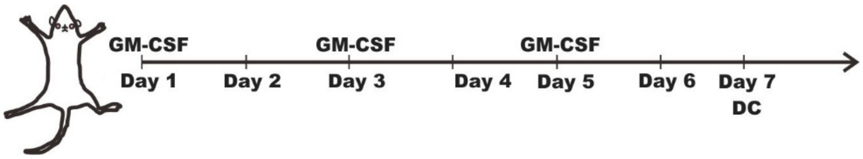

Figure S15. *In vitro* DC differentiation scheme of bone marrow cells cultured for 7 days with GM-CSF added every two days.

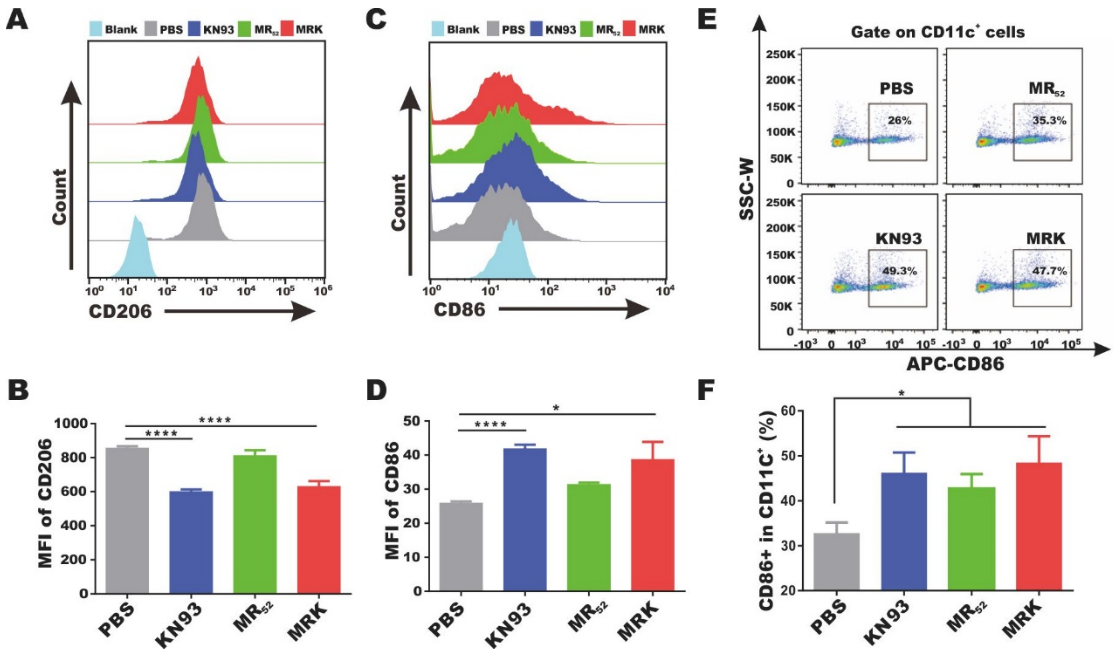

Figure S16. MRK hydrogel reprogrammed M2 toward M1 phenotype and promoted DC maturation. (A-D) IL-4 and IL-13 polarized BMDMs were treated with PBS, KN93, MR_{52}, or MRK hydrogel for 24 h. The expression of CD206 and CD86 was measured by flow cytometry. Data are means ± SEM (n = 3). **(E, F)** Evaluation of the expression levels of the DC mature markers CD86 upon various treatments as indicated. Data are presented as the mean ± SEM (n = 3).

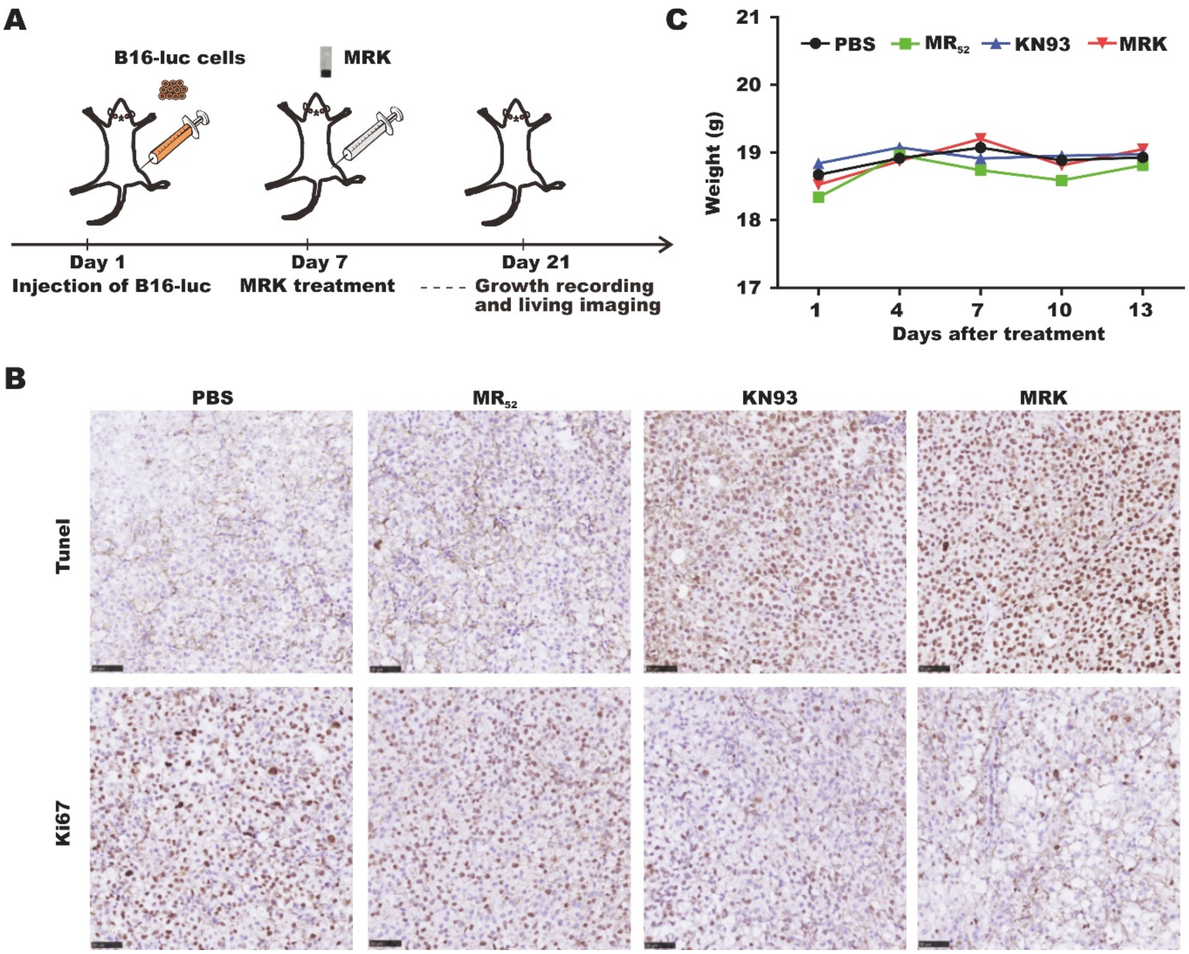

Figure S17. *In vivo* therapeutic effects of the MRK hydrogel. (A) Schematic illustration of the MRK hydrogel treatment schedule for subcutaneously implanted B16-luc melanoma model. **(B)** Representative histological examinations of the dissected tumors using the TUNEL assay and Ki67 staining (scale bar, 50 μm). **(C)** Quantification of tumor weights in each group. Data are presented as the mean ± SEM (n = 7-8).

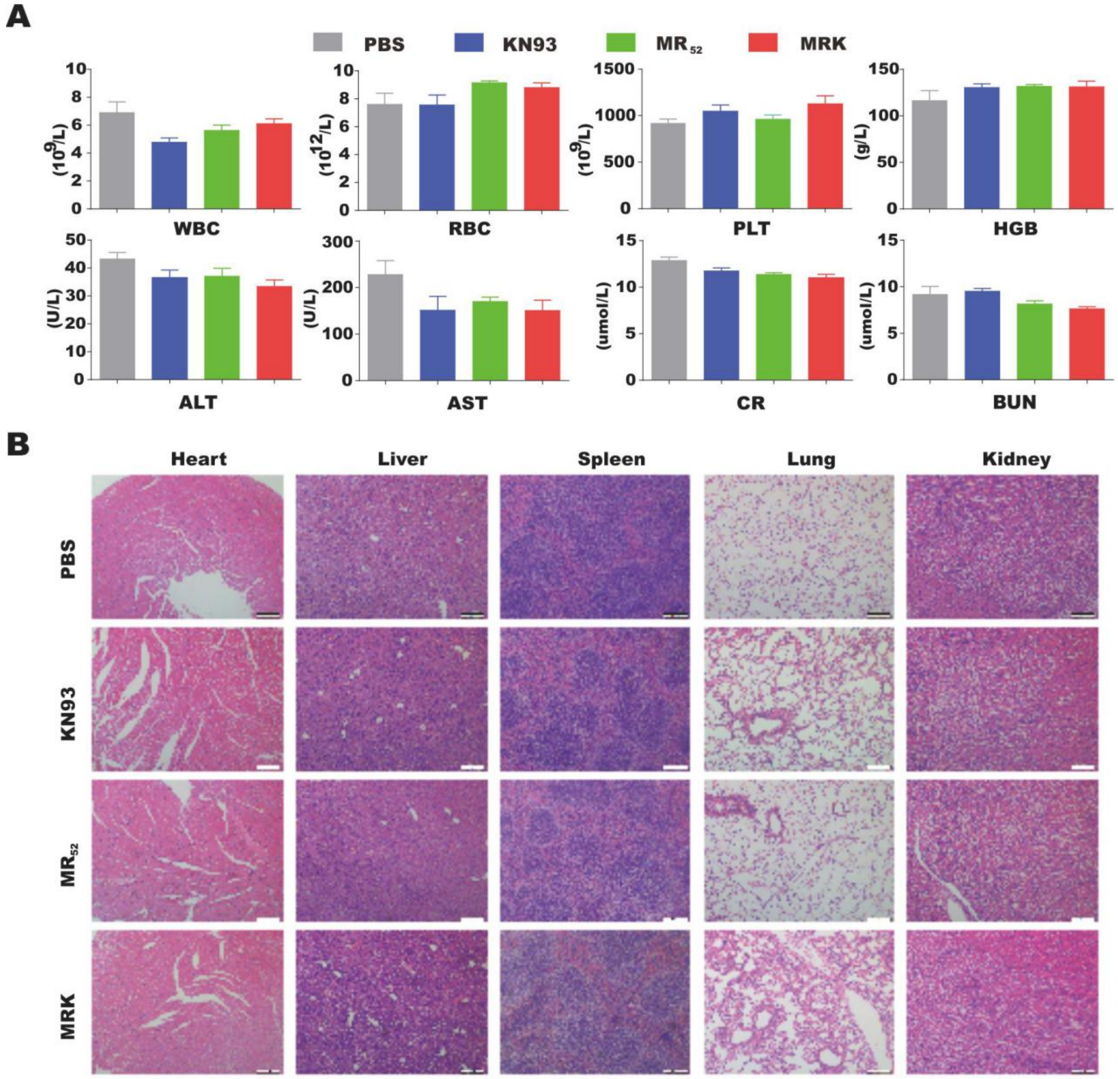

Figure S18. Biocompatibility of hydrogel *in vivo*. (A) Hemanalysis was performed on blood withdrawn from mice on day 8 post drug treatment. Data are means ± SEM (n = 8). **(B)** H&E stained micrographs of the major organs collected from mice injected with PBS, KN93, MR_{52}, or MRK hydrogel. (scale bar: 100 μm).

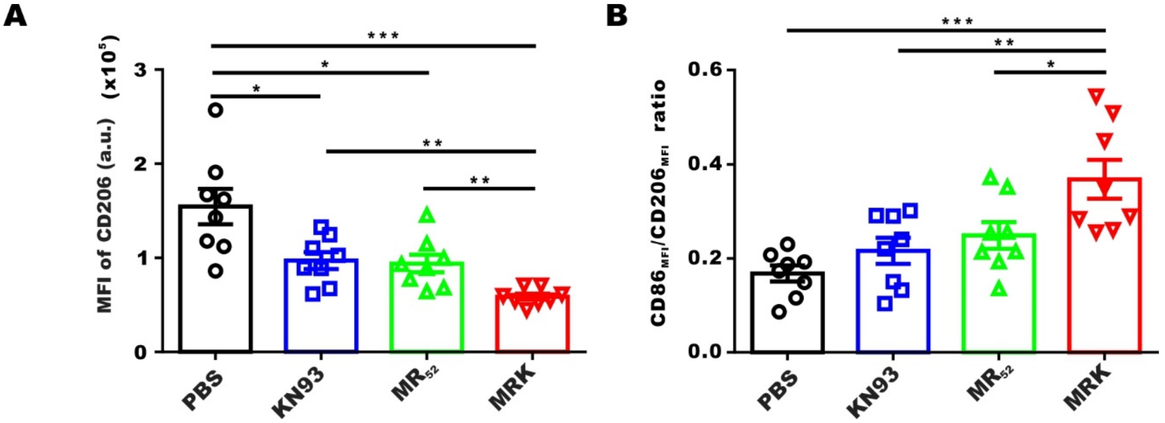

Figure S19. Mean fluorescence intensity (MFI) of CD206 in TAMs (ZIR$^-$CD11b$^+$F4/80$^+$) from various treatment groups **(A)**. MFI of CD86/ MFI of CD206 ratio in TAMs from various treatment groups **(B)**

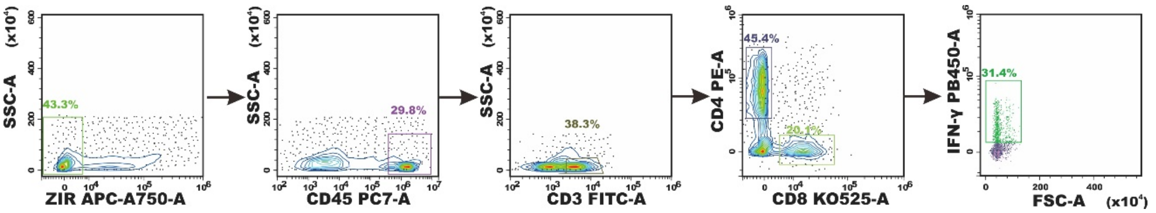

Figure S20. Flow cytometry gating strategy for the detection ratio of Th1 cells and CTLs.

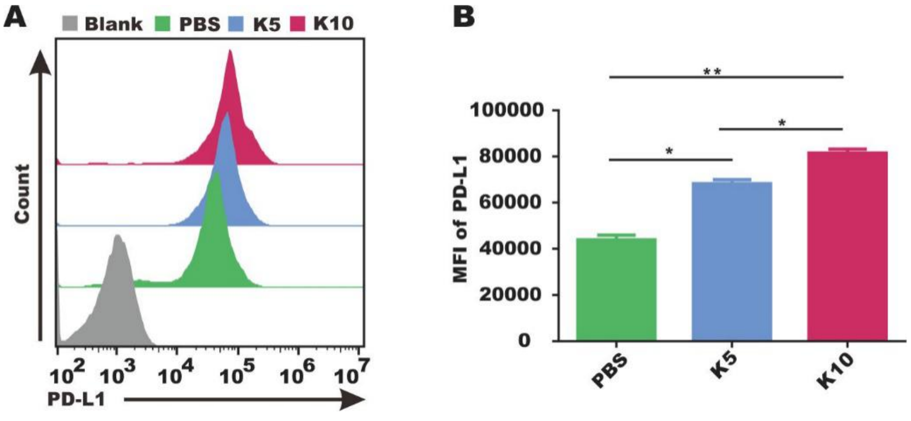

Figure S21. PD-L1 expression detection in BMDMs after various treatments as indicated by flow cytometry. Data are presented as the mean ± SEM (n = 3).

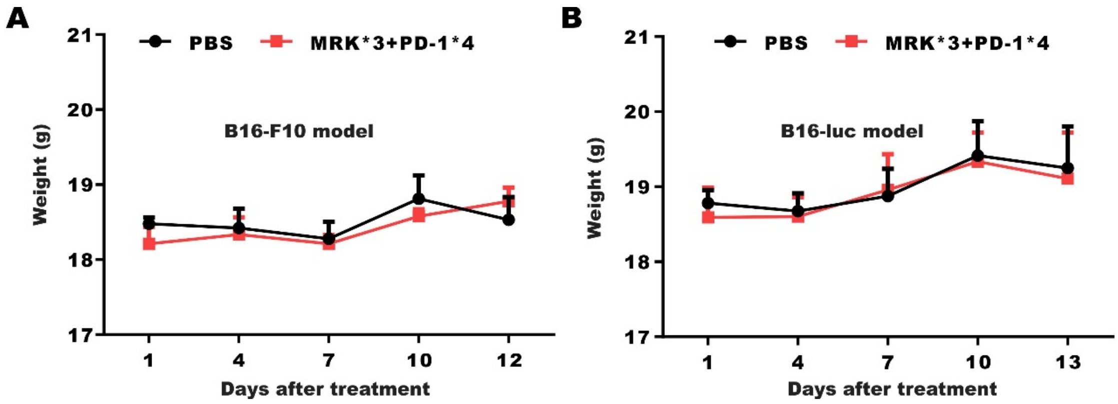

Figure S22. Mice weights measurement at indicated time points in B16F10 or B16-luc melanoma model.

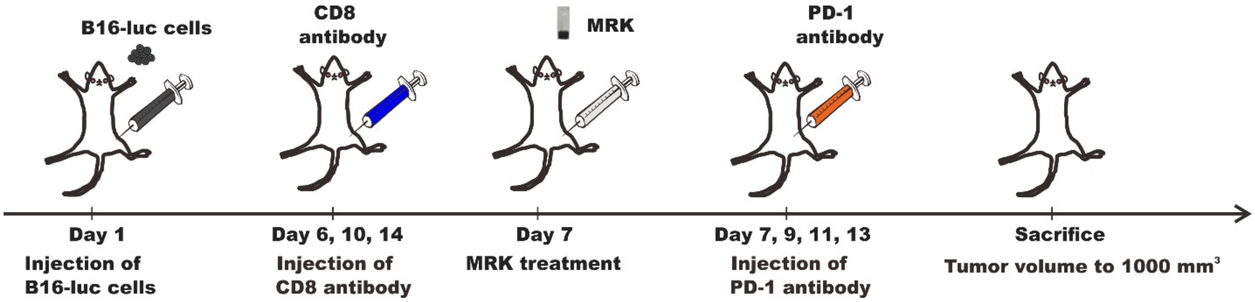

Figure S23. Schematic illustration of anti-CD8 neutralizing antibody and/or MRK plus anti-PD-1 treatment schedule for the B16-luc melanoma model.

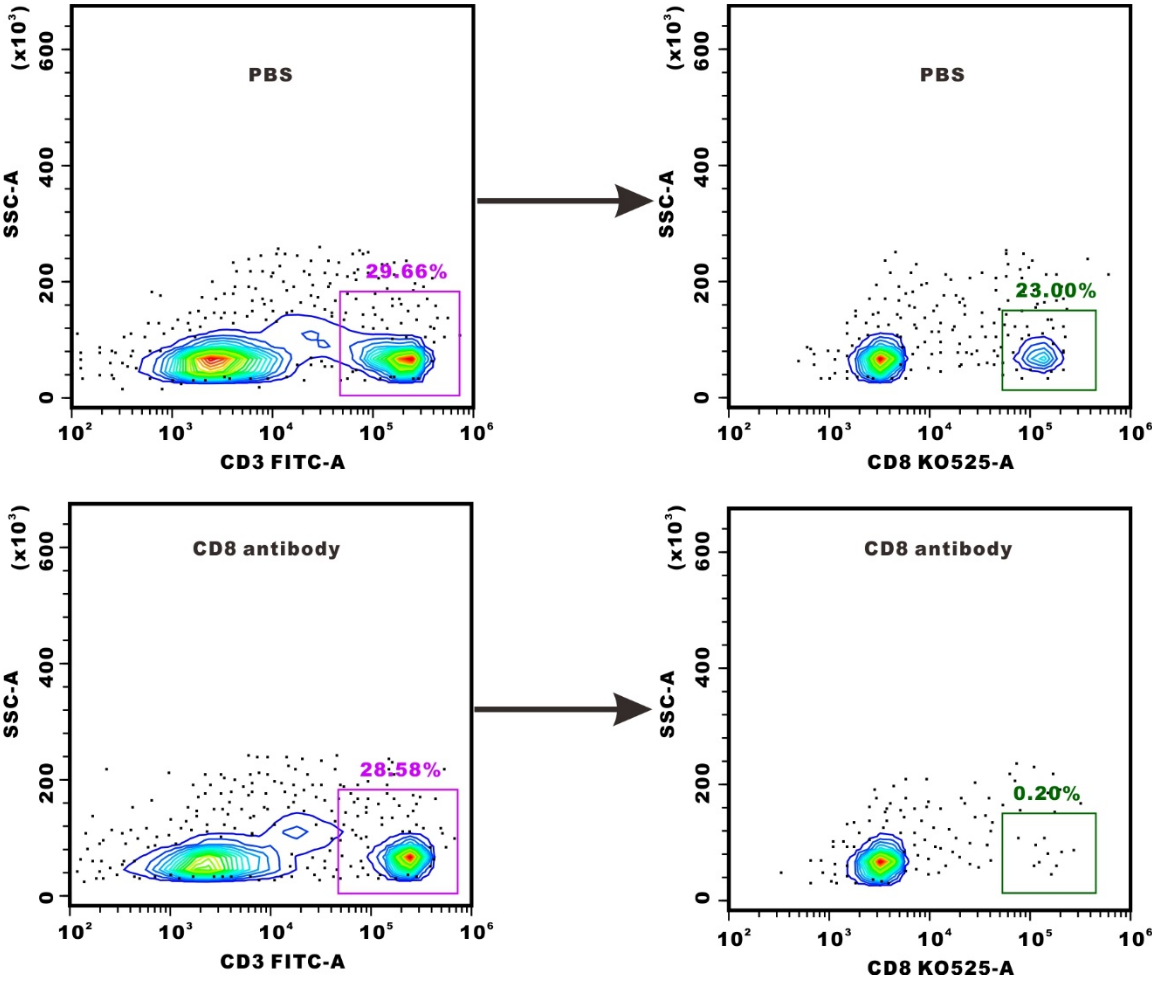

Figure S24. Verification of clearance efficiency of CD8 neutralizing antibodies in peripheral blood of C57BL/6 mice.

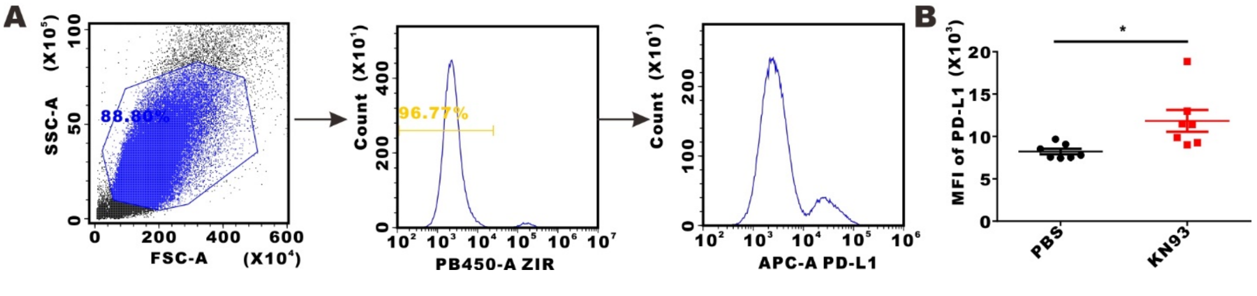

Figure S25. Detection of PD-L1 expression in H22-luc ascites microenvironment (ZIR$^-$ living cells) after KN93 treatment by flow cytometry. **(A)** Flow cytometry gating strategy for the

detection of PD-L1 expression in H22-luc ascites microenvironment; **(B)** Mean fluorescence intensity (MFI) of PD-L1 in ascites microenvironment (ZIR$^-$ living cells) after KN93 treatment (n = 7).

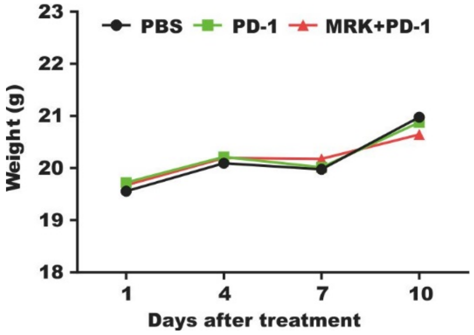

Figure S26. Mice weights measurement at indicated time points after various treatments in H22 hepatoma ascites model.

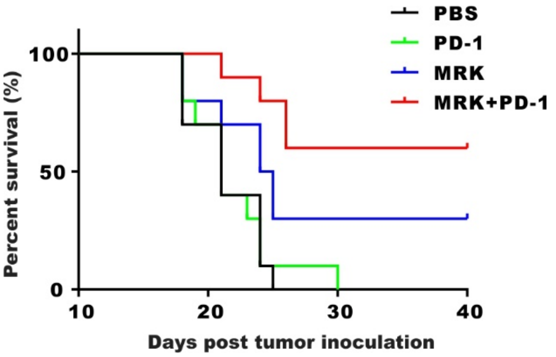

Figure S27. The long-term survival was recorded according to Kaplan–Meier analysis that BALB/c mice (n = 10) with H22-luc hepatoma ascites were treated with PBS, PD-1, MRK, and MRK plus PD-1 respectively.
